# Biosynthesis of silver nanoparticles from *Syzygium cumini* leaves and their potential effects on odontogenic pathogens and biofilms

**DOI:** 10.3389/fmicb.2022.995521

**Published:** 2022-09-29

**Authors:** Wagner Luis de Carvalho Bernardo, Marcelo Fabiano Gomes Boriollo, Caroline Coradi Tonon, Jeferson Júnior da Silva, Mateus Cardoso Oliveira, Fernando Cruz de Moraes, Denise Madalena Palomari Spolidorio

**Affiliations:** ^1^Department of Physiology and Pathology, School of Dentistry, São Paulo State University, Araraquara, Brazil; ^2^Department of Oral Diagnosis, Dental School of Piracicaba, State University of Campinas, Piracicaba, Brazil; ^3^School of Chemistry, Federal University of São Carlos, São Carlos, Brazil

**Keywords:** silver nanoparticles, *Syzygium cumini*, antimicrobial activity, antibiofilm activity, normal oral keratinocytes spontaneously immortalized, odontogenic pathogens

## Abstract

This study analyzed the antimicrobial and antibiofilm action and cytotoxicity of extract (HEScL) and silver nanoparticles (AgNPs-HEScL) from *Syzygium cumini* leaves. GC–MS, UV–Vis, EDX, FEG/SEM, DLS and zeta potential assays were used to characterize the extract or nanoparticles. Antimicrobial, antibiofilm and cytotoxicity analyses were carried out by *in vitro* methods: agar diffusion, microdilution and normal oral keratinocytes spontaneously immortalized (NOK-SI) cell culture. MICs of planktonic cells ranged from 31.2–250 (AgNPs-HEScL) to 1,296.8–10,375 μg/ml (HEScL) for *Actinomyces naeslundii*, *Fusobacterium nucleatum*, *Staphylococcus aureus*, *Staphylococcus epidermidis*, *Streptococcus mutans*, *Streptococcus oralis*, *Veillonella dispar*, and *Candida albicans*. AgNPs-HEScL showed antibiofilm effects (125–8,000 μg/ml) toward *Candida albicans*, *Streptococcus mutans* and *Streptococcus oralis*, and *Staphylococcus aureus* and *Staphylococcus epidermidis*. The NOK-SI exhibited no cytotoxicity when treated with 32.8 and 680.3 μg/ml of AgNPs-HEScL and HEScL, respectively, for 5 min. The data suggest potential antimicrobial and antibiofilm action of HEScL, and more specifically, AgNPs-HEScL, involving pathogens of medical and dental interest (dose-, time- and species-dependent). The cytotoxicity of HEScL and AgNPs-HEScL detected in NOK-SI was dose- and time-dependent. This study presents toxicological information about the lyophilized ethanolic extract of *S. cumini* leaves, including their metallic nanoparticles, and adds scientific values to incipient studies found in the literature.

## Introduction

*Syzygium* tree (of family Myrtaceae) comprises about 1,100 species. It is native to tropical areas – tropical America and Australia – extending across the Pacific region to Africa, Madagascar, South and East Asia. The plants in this family are rich in volatile oils, produce edible fruits and have often been used for medicinal purposes. *Syzygium cumini* Lam. is one of the best known species and has been popularly called jambolão (Brazil), black plum, java plum, Indian berry, Portuguese plum, Malabar plum, purple plum, Jamaica plum, and damson (Ayyanar and Subash-Babu, 2012). In alternative medicine, all parts of a black plum are used as a liver tonic to treat diabetes, blisters in mouth, cancer, colic, diarrhea, digestive disorders, dysentery, hemorrhoids, acnes, and stomachache (Jain, 1991), in addition to being used for scalp mycosis lotion and as a blood strengthener, antioxidant, anti-inflammatory, antimicrobial, anti-HIV, antifungal agent, a substance to eliminate nitric oxide and free radicals, and an antidiarrheal, anti-fertility, anorexigenic, gastroprotective, and radioprotective agent (Sagrawat et al., 2006). Ashes from black plum leaves have been reported to strengthen teeth and gums (Kiritikar and Basu, 1987), while the leaf juice alone or combined with other substances (e.g., other plants, goat milk, honey, etc.) have been used to treat dysentery (Nadkarni, 1976).

Plants produce functional biomolecules that actively reduce metal ions (green synthesis). All parts of plants and their bioactive compounds (polyphenols, alkaloids, flavonoids, and terpenes) can be used to efficiently synthesize nanoparticles. Then, a synthesis process involves three key factors: the reducing agent, the reaction medium and the stabilizing agent (Thill et al., 2006; Husen and Siddiqi, 2014). Biosynthesis of silver nanoparticles (AgNPs) is a ‘bottom-up’ method that mainly involves oxidation and reduction reactions. The main advantage of using plant extract in AgNPs synthesis is the fact that they are readily available, safe, and, in most cases, non-toxic, besides having a wide variety of chemical substances (terpenoids, flavonoids, ketones, aldehydes, amides and carboxylic acids; Prabhu and Poulose, 2012). Among metal nanoparticles, AgNPs are the most widely used as an antimicrobial agent (bacteria, fungi and viruses). Although this antimicrobial activity has been reported to be dependent on the particle size, small diameters of nanoparticles have a greater antimicrobial effect than larger ones (Siddiqi et al., 2018). Additionally, there is evidence that bacteria develop less resistance against AgNPs than against antibiotics (Chernousova and Epple, 2013). However, some controversial aspects should be discussed: the definition and determination of the minimum inhibitory concentration, the emergence of resistant strains, bactericidal or bacteriostatic mechanisms of AgNPs, the cytotoxic effects on humans, and the destruction of planktonic microbial cells and biofilm only (Sheng and Liu, 2011; Majdalawieh et al., 2014).

Planktonic microbial species in the oral cavity can establish reversible links with acquired enamel pellicle (e.g., salivary proteins, proteins, carbohydrates and lipids not derived from saliva) through physical adhesion, electrostatic attractions or predominantly chemical interactions (Hojo et al., 2009). The main pioneer bacterial genera of dental biofilm include *Actinomyces* sp., *Streptococcus* sp., *Haemophilus* sp., *Capnocytophaga* sp., *Veillonella* sp., and *Neisseria* sp., which adhere to the tooth surface and support other species in colonization and competition events (Hojo et al., 2009). Other microorganisms such as *Fusobacterium nucleatum*, *Treponema* sp., *Tannerella forsythensis*, *Porphyromonas gingivalis*, and *Aggregatibacter actinomycetemcomitans* among others can be found in the maturation stage of the dental biofilm. These species recognize and adhere to glycoprotein receptors present in pioneer microorganisms, creating complex structures of dental biofilms that favor microbial interaction and adaptation processes (Kreth et al., 2005). At this stage of dental biofilm development, dispersion of single cells or microbial cell groups can occur by erosion, scaling and spreading (Parsek and Singh, 2003) as a result of shortage of nutrients at the site or due to host defense events (i.e., saliva shear stress; Parsek and Singh, 2003). Other microorganisms of medical interest are involved in serious human infections and spread of strains that are resistant to antimicrobials, such as *Staphylococcus aureus* and *Candida albicans* (Chung and Toh, 2014; Suleyman and Alangaden, 2016).

Epithelial cells lining the oral cavity and epidermis usually express high levels of cytokeratins and are called keratinocytes (Presland and Dale, 2000). The oral keratinocytes build a keratinized and non-keratinized stratified epithelium, depending on the location in the oral cavity (Presland and Dale, 2000). The oral epidermal keratinocytes are organized in several layers and subject to a constant regenerative process. In general, proliferation occurs in the basal layer of the keratinocytes fixed to the underlying basement membrane; cells which suffer terminal differentiation while migrating through the suprabasal layers are eliminated from the tissue surface (Watt, 1989). On the other hand, human oral keratinocytes are highly sensitive to toxic substances, and *in vitro* studies involving their cell cultures exposed to test substances, including plants and their derivatives (Lukandu et al., 2008; Costea et al., 2010; Sheng et al., 2018), add information about the potential damages to the human oral epithelium.

Therefore, this study aimed to investigate the potential of biological activities of plants and green synthesis of metal nanoparticles, particularly of hydroalcoholic extract and silver nanoparticles from *S. cumini* leaves, by evaluating the antimicrobial activity, the *in vitro* formation of single and mixed biofilms (oral bacteria and yeast), and the *in vitro* cytotoxic activity. The phytochemical characteristics and green synthesis (formation, structure, size distribution and morphology of nanoparticles) were also evaluated.

## Materials and methods

### Plant extract

Plant samples (leaves) of *Syzygium cumini* (L.) Skeels (synonym: *Eugenia jambolana* Linn.) were harvested from January to May 2017 (corresponding to the fruiting and flowering seasons), in the municipality of Araraquara, São Paulo, Brazil (Latitude: −21.7946, Longitude: −48.1766; 21°47′41″ South, 48°10′36″ West). The samples were washed with sterile distilled water, dried at 50°C, crushed, and the resulting powder was mixed with 70% ethanol in the proportion of 10% (w/vol). The mixtures were stocked with ambient seasoning for 25 days, protected from light and filtered (Whatman filter paper No. 3; de Carvalho Bernardo et al., 2021).

Aliquots (0.5 L) of the filtered hydroalcoholic extract of the *S. cumini* leaves (HEScL) were submitted to the solvent removal process by rotary evaporation using Rotary Evaporator RV 10 Control V (40 rpm, IKA^®^ Works, Inc., United States), coupled to bath heating systems (40°C, Heating Baths HB10, IKA^®^ Works, Inc., United States), a vacuum pump (175 mbar, Chemistry diaphragm pump MD 1C, VACUUBRAND GMBH + CO KG, Wertheim, Germany), a recirculator and water distiller (10°C, Ultra Thermostatic Digital Bath, SPLABOR, Presidente Prudente, São Paulo, Brazil) and an evaporation bottle (RV 10.85 Evaporation Flask, NS 29/32-2l, IKA^®^ Works, Inc., United States). The products were transferred to 0.5 L reaction vials (SCHOTT^®^ DURAN^®^) and kept at −20°C for 24 h to evaluate the freezing and efficacy of the solvent evaporation process (Boriollo et al., 2020). Then, aliquots (20 ml) of the products were lyophilized (0.12 mbar at −50°C; Lyophilizer model Alpha 1–2 LDPlus, Martin Christ Gefriertrocknungsanlagen GmbH^©^, Germany), and their masses were determined (Electronic Analytical Balance AUW-220D, Shimadzu Corp., Kyoto, Japan).

### Gas chromatography – mass spectrometry

Lyophilized products of *S. cumini* extracts (HEScL) were dissolved with ethanol. Dispersions were filtered and analyzed by GC–MS (Aligent 5975C TAD Series GC/MSD System, ^©^Aligent Technologies, Inc., CA, United States) using the following chromatographic conditions: (i) Sample: injected volume of 2.0 μl; (ii) Column: HP-5MS, 5% difenil, 95% dimetil polisiloxano (30 m × 0.25 mm × 0.25 μm); (iii) Drag gas: He (99.9999) → 1 ml/min; (iv) Gun: 280°C, Split 1:1; (v) Oven: 50°C (2 min) → 250°C (5°C/1 min); 250°C (10 min); and (vi) Detector: Linear Quadruple Mass Spectrophotometer, Ionization Source (Electron Impact → 70 eV), Scanning Mode (0.5 seg/scan), Masses Range [33–500 Dalton (u)], Transfer Line (280°C) and Filament (off at 7.0 min). The mass spectral database (NIST 11) and the AMDIS program (Automated Mass Spectral Deconvolution and Identification System) were used to identify compounds detected in chromatograms (Boriollo et al., 2020; de Carvalho Bernardo et al., 2021).

### Green synthesis of AgNPs-plant extracts

Lyophilized hydroalcoholic extracts of *S. cumini* (HEScL) were used in the synthesis of silver nanoparticles (AgNPs-HEScL). The reaction mixture was prepared by adding 10 ml of the extract solution at 10% (0.1 g/ml) in 990 ml of 1 mM AgNO_3_ solution (type 1 water, Milli Q) inside 1 L laboratory flasks (BOECO GERMANY, Germany). The mixtures were kept under constant agitation for 2 h at 90°C (Khan et al., 2014; de Carvalho Bernardo et al., 2021). Subsequently, these mixtures were cooled down, centrifuged and washed (type 1 water) three times at 12,000 × *g* for 10 min at −4°C (Centrifuge 5,810 R Eppendorf, Hamburg, Germany). The sediments obtained were washed twice with type 1 water at 12,000 × *g* for 10 min at −4°C, transferred to 2 ml microtubes (Axygen Brasil^®^, Corning Inc.) and dehydrated at 65°C overnight (Dryblock, Eppendorf, Hamburg, Germany). The tubes containing AgNPs-HEScL were stored at −70°C until the moment of use.

### Ultraviolet–visible spectroscopy

The synthesis of AgNPs-HEScL was monitored by UV–Vis spectroscopy (spectrum 200–800 nm), operating at a 1 nm resolution (Thermo Scientific NanoDrop^®^ 2000c Spectrophotometer, Wilmington, DE, United States). Stock solutions were prepared by dispersion of nanoparticles in type 1 water at 1:10 (as a reference solvent), followed by sonication for 20 min. These samples were diluted again at a ratio of 1:5 for UV–Vis evaluation (Khan et al., 2014; Kota et al., 2017; Siddiqi et al., 2018; de Carvalho Bernardo et al., 2021).

### Energy-dispersive X-ray spectrophotometry

The chemical nature of AgNPs-HEScL surface elements was investigated using energy-dispersive X-ray spectrometry (EDX) and scanning electron microscopy (SEM). Aliquots of 10 μl of the AgNPs-plant extract solution were applied to pieces of epoxy resin with double-sided carbon conductive tape and coated with carbon spray (Desk II, Denton Vacuum, Moorestown, NJ, United States). Each treated surface was scanned using energy-dispersive X-ray spectrometry (EDX – Vantage Digital Microanalysis, Noran instruments, Middleton, WI, United States) with a scanning electron microscope (SEM; JSM5600 LV; JEOL, Tokyo, Japan) operating at 15 kV and at a working distance of 20 mm, 30° and 5 iterations. The chemical elements of AgNPs-plant extract were quantified, and the entire area displayed at 500× magnification was evaluated (Murillo-Gómez et al., 2018; Siddiqi et al., 2018; de Carvalho Bernardo et al., 2021).

### Field electron gun – scanning electron microscopy

For the ultrastructural and morphological evaluation of AgNPs-HEScL, aliquots of 25 μl of the nanoparticles were deposited on a carbon surface adhered to a metal cylinder (∅ 30 mm × 10 mm) and stored in a desiccator connected to a vacuum pump (Fisatom 802 – Aspirator pump model 7049-50 Cole-Parmer Instrument Company) for 24 h. The nanoparticle images were obtained using a scanning electron microscope connected to a field electron gun (FEG-SEM, Carl Zeiss Microscopy-Gemini Supra 35, software), and analyzed in terms of shape, size and arrangement (Siddiqi et al., 2018; de Carvalho Bernardo et al., 2021).

### Dynamic light scattering and zeta potential

AgNPs-HEScL were diluted in type 1 water (2 mg/ml). Aliquots of 1 to 1.5 ml of the nanoparticles were dispensed in quartz cuvettes (Malvern Analytical – DTS1070) and submitted to analysis on a Zetasizer Nano – ZS – ZEN 3600 equipped with a red He-Ne laser and at a wavelength of 632.8 nm (MALVERN). The results of size, relative diversity and zeta potential of the nanoparticles were obtained with Zetasizer software (Siddiqi et al., 2018; de Carvalho Bernardo et al., 2021). The results of the zeta potential and colloidal stability of the system were interpreted as previously reported (Patel and Agrawal, 2011; Bhattacharjee, 2016; de Carvalho Bernardo et al., 2021). A minimum value of ±30 mV is required for electrostatically stabilized nanosuspensions, while a minimum of ±20 mV is required for steric stabilization.

### Microorganisms and growth condition

The bacterial strains of *Actinomyces naeslundii* ATCC^®^ 12104D-5™, *Fusobacterium nucleatum* subsp. *nucleatum* ATCC^®^ 25586D-5™, *Staphylococcus aureus* ATCC^®^ 25923, *Staphylococcus epidermidis* ATCC^®^ 12228, *Streptococcus mutans* ATCC^®^ 25175D-5™, *Streptococcus oralis* ATCC^®^ 35037™ and *Veillonella dispar* ATCC^®^ 17748™ and the yeast strain of *Candida albicans* ATCC^®^ 90028™ were submitted to susceptibility testing against HEScL and AgNPs-HEScL. Prior to the tests, anaerobic bacteria were grown in blood agar (AS Oxoid, Hampshire, United Kingdom) supplemented with 5 mg/ml of hemin (Sigma-Aldrich Cat. # H9039, Merck KGaA, Darmstadt, Germany), 1 mg/ml of menadione (Sigma-Aldrich Cat. # M5625, Merck KGaA, Darmstadt, Germany) and 5% sterile sheep blood (E&O Laboratories, Scotland, United Kingdom) at 37°C for 48 h in anaerobic conditions (80% N_2_, 10% H_2_ and 10% CO_2_; MiniMacs Anaerobic Workstation, Don Whitley Scientific, Shipley, United Kingdom). Staphylococci and Streptococci were grown in BHI agar at 37°C for 24 h under conditions of aerobiosis and 10% CO_2_, respectively (Water Jacketed CO_2_ Incubator, Thermo Fisher Scientific Inc., Waltham, Massachusetts, United States). Yeasts were grown in SDA agar (Sabouraund Dextrose Agar-Oxoid, Hampshire, United Kingdom) at 35°C for 24 h in aerobic conditions (CLSI, 2008, 2009, 2012a,b,c,d, 2018; Boriollo et al., 2020; de Carvalho Bernardo et al., 2021).

### Antimicrobial susceptibility tests using disk diffusion

*In vitro* antimicrobial susceptibility profiles of HEScL and AgNPs-HEScL against bacterial and yeast strains were determined using the disk diffusion method, observing the guidelines of the Clinical and Laboratory Standards Institute (CLSI, 2008, 2009, 2012b,c, 2018), with some adaptations (de Carvalho Bernardo et al., 2021). Initially, aqueous solutions of HEScL and AgNPs-HEScL were prepared using aqueous solvent (type 1 water) and sterilized by filtration (Millipore Corporation, hydrophilic Durapore^®^ PVDF, 0.22 μm, ∅ 47 mm) at an initial concentration of 166,000 μg/ml for HEScL (16.6% w/v) and 8,000 μg/ml for AgNPs-HEScL (0.8% w/v). Prior to antimicrobial sensitivity tests, paper disks (white sterile disks, ∅ 6 mm and 0.8 mm thick, #570640, Laborclin Produtos para Laboratórios, Pinhais, PR, Brazil) were saturated (6 μl) with solutions of extracts and nanoparticles.

Recently grown microorganisms were suspended again in 5 ml of sterile saline solution (145 mM NaCl) and adjusted to 0.5 turbidity on the McFarland scale (bacteria: 1–4 × 10^8^ CFU/ml; yeasts: 1–5 × 10^6^ CFU/ml) or equivalent to a transmittance of 79.5%–83.2% using a spectrophotometer (Thermo Scientific Multiskan GO UV/Vis, Microplate and Cuvette Spectrophotometer, Waltham, MA, United States) at wavelengths of 625 nm and 530 nm for bacteria and yeasts, respectively (*T* = 79.5–83.2% → A_625 nm/530 nm_ = 2 – log_10_%T → A_625 nm/530 nm_ = 0.100–0.080). This suspension was stirred for 15 s (vortex) and plated (Spread-Plate method) in sterile agar medium previously prepared in a Petri dish (150 × 15 mm; 50 ml culture medium per plate):

- Brucella Agar (BD BBL™ Brucella Agar with 5% Sheep Blood, Hemin and Vitamin K1, Germany) for *A. naeslundii*, *F. nucleatum* subsp. *nucleatum* and *V. dispar*;
- Mueller Hinton Agar, pH 7.2 to 7.4 (Mueller Hinton Broth No.2 Control Cations, Himedia) for *S. aureus* and *S. epidermidis*;
- Mueller Hinton Agar supplemented with 2% glucose (Mueller Hinton Agar, 2% Glucose with Methylene blue, Himedia) for *C. albicans*;
- Mueller Hinton Agar supplemented with 5% horse blood (BD Mueller Hinton Agar, Becton Dickinson GmbH, Heidelberg, Germany) for *S. mutans* and *S. oralis*.

These plates were kept at room temperature for 15 min for full absorption of the inoculum in the culture medium. Then, the disks containing extracts and nanoparticles were aseptically placed on the surface of the inoculated culture medium, and the plates were incubated inversely at 35 and 37°C for 24 h under specific environmental conditions for each microorganism. These tests were conducted in triplicate test systems and the interpretation of results was performed from the zone of microbial growth inhibition (halo diameter in mm). Disks containing aqueous solutions of 0.12% chlorhexidine (12 mg/ml; CAS # 18472-51-0; Chlorhexidine digluconate, SUPELCO Cat. # PHR1294, Merck KGaA, Darmstadt, Germany) and 0.00025% fluconazole (25 μg/ml; triazole; CAS # 86386-73-4; Sigma-Aldrich Cat. # F8929, Merck KGaA, Darmstadt, Germany) were used as positive control (inhibition zone), while disks containing sterile type 1 water were used as negative control (no inhibition zone). Chlorhexidine is often used in surgical procedures as pre-operative skin antiseptics and recommended for surgical sites disinfections (Alexander et al., 2011; Zhang et al., 2017; Silva et al., 2018). It has also been extensively used in dental practice to treat oral disease and reduce plaque formation due to its antimicrobial effects against oral pathogens (Brookes et al., 2021).

### Antimicrobial susceptibility tests using broth dilution

The minimum inhibitory concentration (MIC) of HEScL and AgNPs-HEScL with bacterial and yeast strains was determined by broth microdilution, observing the guidelines of the Clinical and Laboratory Standards Institute (CLSI, 2008, 2012a,d, 2018), with some adaptations (Silva et al., 2018; Boriollo et al., 2020; de Carvalho Bernardo et al., 2021). These tests were performed in triplicate test systems using microdilution plates with multiple wells (96-well cell culture microplates, Corning Inc., NY, United States) containing 100 μl/well of sterile culture medium supplemented with HEScL or AgNPs-HEScL, including the microbial inoculum. The concentrations of HEScL used in the test ranged from 166,000 μg/ml to 324.2 μg/ml of culture medium, whereas those of AgNPs-HEScL varied between 8,000 μg/ml and 15.6 μg/ml of culture medium.

The recently grown microorganisms were adjusted to a transmittance of 79.5%–83.2% using a spectrophotometer (Thermo Scientific Multiskan GO UV/Vis, Microplate and Cuvette Spectrophotometer, Waltham, MA, United States) at wavelengths of 625 nm and 530 nm for bacteria and yeasts, respectively (*T* = 79.5–83.2% → A_625 nm/530 nm_ = 2 – log_10_%*T* → A_625 nm/530 nm_ = 0.100–0.080). The culture media employed were as follows:

- Brucella broth (BD BBL™ Brucella Broth with 5% Sheep Blood, Hemin and Vitamin K1, Germany) for *A. naeslundii*, *F. nucleatum* subsp. *nucleatum*, and *V. dispar*;
- Mueller Hinton broth, pH 7.2–7.4 (Mueller Hinton Broth No. 2 Control Cations, Himedia) for *S. aureus* and *S. epidermidis*;
- Cation-adjusted Mueller Hinton broth, supplemented with horse blood lysed at 5% for *S. mutans* and *S. oralis*;
- RPMI-1640 culture medium buffered with MOPS [1.04% w/v RPMI-1640 (Sigma-Aldrich Cat. # R7388, Merck KGaA, Darmstadt, Germany), 165 mM MOPS (Sigma-Aldrich Cat. # M3183, Merck KGaA, Darmstadt, Germany), glucose 2% w/v, pH 7.0 adjusted with 1 M NaOH at 25°C and sterilized by filtration] for *C. albicans*.

Afterwards, the microdilution plates were incubated at 35 or 37°C for 24–48 h under specific environmental conditions for each microorganism. These tests were performed in triplicate test systems and the interpretation of breakpoints was based on the guidelines of the Clinical and Laboratory Standards Institute. In addition, colorimetric assays based on resazurin biotransformation systems by bacteria (Resazurin sodium salt, Sigma-Aldrich Cat. # R7017, Merck KGaA, Darmstadt, Germany; Martin et al., 2003) and yeast TTZ (2, 3,5-Triphenyltetrazolium chloride, Sigma-Aldrich Cat. # T8877, Merck KGaA, Darmstadt, Germany; Silva et al., 2018; de Carvalho Bernardo et al., 2021) were performed in order to determine the minimum inhibitory concentrations of HEScL and AgNPs-HEScL.

### Minimum bactericidal and fungicidal concentration

The minimum bactericidal and fungicidal concentrations (MBC and MFC, respectively) of HEScL and AgNPs-HEScL was determined according to a previously proposed methodology (Cantón et al., 2003; Boriollo et al., 2020; de Carvalho Bernardo et al., 2021). For each microbial strain, aliquots of 50 μl of the total well volume corresponding to the value ≥MIC were homogenized with a pipette, sown in Petri dishes containing specific solid culture media for each species studied and incubated at 35°C–37°C for 24–48 h under specific environmental conditions for each species. MBC or MFC was the lowest concentration of HEScL or AgNPs-HEScL able to eliminate ≥99.9% of the final inoculum (0.05–0.25 colony).

### *In vitro* cytotoxicity tests

Prior to cytotoxicity tests, HEScL and AgNPs-HEScL were diluted in DMEM/F-12 culture medium (Sigma-Aldrich Cat. # D6421, Dulbecco’s Modified Eagle’s Medium/Nutrient Mixture F-12 Ham, Merck KGaA, Darmstadt, Germany), supplemented with 10% FBS (Fetal Bovine Serum, Sigma-Aldrich Cat. # F0320, Merck KGaA, Darmstadt, Germany), 1% penicillin (100 IU/ml; CAS number 69-57-8, Penicillin G sodium salt, Sigma-Aldrich Cat. # P3032, Merck KGaA, Darmstadt, Germany), 1% streptomycin (100 μg/ml; CAS number 3810-74-0, Streptomycin sulfate salt, Sigma-Aldrich Cat. # S9137, Merck KGaA, Darmstadt, Germany) and 1% glutamine (2 mmol/L; L-Glutamine, Sigma-Aldrich Cat. # G6392, Merck KGaA, Darmstadt, Germany), and sterilized by filtration. The test concentrations used in the cytotoxicity assays were based on the results of the antimicrobial susceptibility assays, considering the minimum and maximum values of MIC (HEScL and AgNPs-HEScL) observed in the population of microorganisms studied:

- HEScL (concentrations equivalent to ≈20.78× > AgNPs-HEScL): 680.3, 1,296.9, 2,593.8, 5,187.5, and 10,375 μg/ml;
- AgNPs-HEScL (concentrations equivalent to ≈20.78× < HEScL): 32.8, 62.5, 125, 250, and 500 μg/ml.

Normal oral keratinocytes spontaneously immortalized (NOK-SI) from gingival tissues of healthy volunteers (National Institute of Dental and Craniofacial Research, Bethesda, MD, United States; Castilho et al., 2010), kept in cryogenic systems at the Biobank of the School of Dentistry, São Paulo State University (FOAr/UNESP, Araraquara, SP, Brazil), were used to assess the cytotoxicity of HEScL and AgNPs-HEScL. NOK-SI were grown in 75 cm^2^ sterile culture flasks (Costar Corp., MA, United States) containing DMEM/F-12 culture medium supplemented with 10% FBS + 1% penicillin (100 IU/ml) + 1% streptomycin (100 μg/ml) + 1% glutamine (2 mmol/L). Cell cultures were produced every 3 days under environmental conditions of 5% CO_2_ at 37°C (Heracell™ 150i CO_2_ Incubators with Stainless-Steel Chambers, Cat. # 51026280, Thermo Fisher Scientific, Waltham, Massachusetts, United States) to obtain an adequate number of cells for the cytotoxicity study. Then, recently grown NOK-SI were seeded (30,000 cells/cm^2^) in sterile 24-well plates (Corning^®^ Costar^®^ TC-Treated Multiple Well Plates, SIGMA Cat. # CLS3526, Merck KGaA, Darmstadt, Germany) under the same nutritional conditions and incubated at 37°C for 48 h and 5% CO_2_. Subsequently, the liquid contents of the wells were carefully replaced with 1 ml of sterile culture media and incubated again at 37°C for 24 h and 5% CO_2_ (Tonon et al., 2018).

For treatments with different concentrations of HEScL and AgNPs-HEScL, the liquid contents of cell culture wells were carefully replaced with 1 ml of HEScL and AgNPs-HEScL solutions (stored at 37°C), followed by incubation at 37°C for 5 and 10 min and 5% CO_2_. After the treatment times, the liquid contents of cell culture wells were carefully aspirated, washed with sterile PBS buffer solution, and incubated at 37°C for 4 h and 5% CO_2_ with AlamarBlue™ Cell Viability Reagent (Invitrogen™, Cat. # DAL1025, Thermo Fisher Scientific, Waltham, Massachusetts, United States) at 10% (vol/vol) in DMEM/F-12. Then, aliquots of 100 μl were transferred from each well to new microdilution plates of 96 wells and analyzed using a microplate reader (BioTek™ Synergy™ H1 Hybrid Multi-Mode All Detection Modes Microplate Readers, Cat. # 11–120-535, Fisher Scientific part of Thermo Fisher Scientific, Waltham, Massachusetts, United States) at wavelengths of 545/590 nm (excitation/emission).

Control components were also included in the cytotoxicity test: 3% hydrogen peroxide (Hydrogen peroxide solution, Sigma-Aldrich Cat. # H1009, Merck KGaA, Darmstadt, Germany) and 0.12% chlorhexidine (Chlorhexidine digluconate, SUPELCO Cat. # PHR1294, Merck KGaA, Darmstadt, Germany) were used as cytotoxic controls, while DMEM/F-12 culture media supplemented with 10% FBS + 1% penicillin (100 IU/ml) + 1% streptomycin (100 μg/ml) + 1% glutamine (2 mmol/L) and without any other test substances were used as cell growth controls. The methodological procedures of cytotoxicity tests were performed in triplicate test systems and using three independent experiments (Tonon et al., 2018).

### Antibiofilm susceptibility tests using AgNPs-HEScL

The antibiofilm action of AgNPs-HEScL was determined with the development of a single biofilm (*C. albicans*) and two mixed biofilms (*S. mutans* + *S. oralis*; *S. aureus* + *S. epidermidis*). For the control tests of biofilm development, recently grown microbial cultures were dispensed (100 μl of *C. albicans* culture; 50 μl of *S. mutans* + 50 μl of *S. oralis*; 50 μl of *S. aureus* + 50 μl of *S. epidermidis*) in microdilution plates with multiple wells (96-well cell culture microplates, Corning Inc., NY, United States) and incubated at 37°C for 90 min under a constant agitation speed of 75 rpm. (Incubadora 430 RDB, Nova Ética Produtos e Equipamentos Científicos Ltda. Vargem Grande Paulista, SP, Brazil), corresponding to the phase of microbial adhesion to the well surface. Soon after that, planktonic cells were carefully removed by washing (2×) with sterile PBS buffer solution (100 mM NaCl, 100 mM NaH_2_PO_4_, pH 7.2). Then, 100 μl of culture media was added to the wells of microplates containing adherent microbial cells [i.e., RPMI 1640 media for yeast and BHI (Brain Heart Infusion Medium, M211-500G, Himedia, Mumbai, India) for bacteria]. These microplates were incubated at 37°C for 24 h under a constant agitation speed of 75 rpm to promote the initial development of single and mixed biofilms. These initial biofilms were carefully washed with sterile PBS solution (2×), followed by the addition of new culture media and incubation at 37°C for 24 h (totaling 48 h) under a constant agitation speed of 75 rpm to promote advanced development of single and mixed biofilms (Bordini et al., 2018; Tonon et al., 2018).

Prior to treatment with the nanoparticles, adherent cells (90 min) and initial (24 h) and advanced (48 h) biofilms were carefully washed (2×) with sterile PBS solution to remove planktonic cells. Subsequently, all these development stages of single and mixed biofilms were exposed to 100 μl aliquots of AgNPs-HEScL solutions (125, 500 and 8,000 μg/ml of RPMI 1640 or BHI medium) and incubated at 37°C for 5 min under a constant agitation of 75 rpm, followed by a new careful washing (2×) with sterile PBS solution. The concentrations tested represented the ≥MIC values (planktonic cells) based on the following criteria: (i) biofilm-embedded microorganisms are up to 1,000-fold more resistant to antibiotics than their planktonic cells (Gilbert et al., 1997), (ii) *C. albicans* biofilm sessile minimum inhibitory concentration (SMIC) values are higher than those assigned for planktonic cells resistant to antifungals (e.g., fluconazole ^7.8×^, amphotericin B ^500×^ and nystatin ^3.9–125× to 15.6–500×^; Bassi and Boriollo, 2020), and (iii) the emergence and progression of dental caries and periodontal diseases are related to the increased resistance to antibiotics by bacterial biofilms (Kouidhi et al., 2015). Aqueous solutions of 0.12% chlorhexidine were used as positive control, that is, control for the inhibition of biofilm formation kinetics. All methodological procedures were performed in triplicate test systems and using three independent experiments.

The cellular metabolic activities present in the three development stages of single and mixed biofilms were monitored by the reduction assay of 2,3-bis(2-methoxy-4-nitro-5-sulfophenyl)-5-[(phenylamino)carbonyl]-2H-tetrazolium hydroxide (XTT), a semi-quantitative analysis measurement (Thein et al., 2007; Tonon et al., 2018). XTT solution (XTT sodium salt, SIGMA Cat. # X4626, Merck KGaA, Darmstadt, Germany) was prepared with type 1 water at a concentration of 1 mg/ml, sterilized by filtration (Millipore Corporation, hydrophilic Durapore PVDF, 0.22 μm, ∅ 47 mm, Cat. # GVWP 047 00) and stored at −80°C until the moment of use. Prior to the test, a recently prepared menadione solution (0.4 mM in acetone; Crystalline Menadione, SIGMA Cat. # M5625, Merck KGaA, Darmstadt, Germany) was added to the XTT solution (final concentration of menadione equivalent to 1 μM). Aliquots of 200 μl of this XTT-menadione solution were distributed to the microdilution plate wells containing adherent cells or initial or advanced biofilms previously treated with AgNPs-HEScL and washed with PBS. These plates were then incubated in the dark at 37°C for 3 h. Then, aliquots of 100 μl were carefully transferred from each well to new microplates, and the soluble formazan product was quantified by spectrophotometry at 492 nm (Multiskan, Ascent 354, Labsystems CE, Lês Ulis, France).

#### SEM and biofilms treated with AgNPs-HEScL

The ultrastructural and morphological evaluations of the development stages of single and advanced mixed biofilms (90 min, 24 and 48 h) exposed to AgNPs-HEScL (125, 500, and 8,000 μg/ml) were performed by scanning electron microscopy. The test conditions were those described above, using LAB-TEK slides (Nunc^®^ Lab-Tek^®^ Chamber Slide™ system, SIGMA Cat. # C7182, Merck KGaA, Darmstadt, Germany). These slides were carefully washed 2× with PBS solution and kept at room temperature until fully dry. These specimens were then fixed in 2% glutaraldehyde (Glutaraldehyde solution, Sigma-Aldrich Cat. # G5882, Merck KGaA, Darmstadt, Germany) in PBS solution for 30 min and dehydrated by sequential washing with ethanol for 10 min (i.e., washing performed 2× at each concentration: 50%, 70%, 90% and absolute ethanol), followed by drying at room temperature. Afterward, the specimens were metallized with gold at 20 Ω for 120 s (Desk V - Standard, Delton Vacuum, LLC, JOEL United States, Inc.) and analyzed on JSM-6610 LV JEOL scanning electron microscope (Tokyo, Japan) at 10 KV and 3,000× magnification (Boni et al., 2016; Tonon et al., 2018).

### Statistical analysis

Data obtained from the antimicrobial susceptibility and antibiofilm and cytotoxicity tests were submitted to analysis of variance (ANOVA) and comparison of means through Tukey’s and Dunnett’s tests (*α* = 0.05) using GraphPad Prism software version 5.0c.

## Results

### GC–MS of HEScL

**Figure 1:**
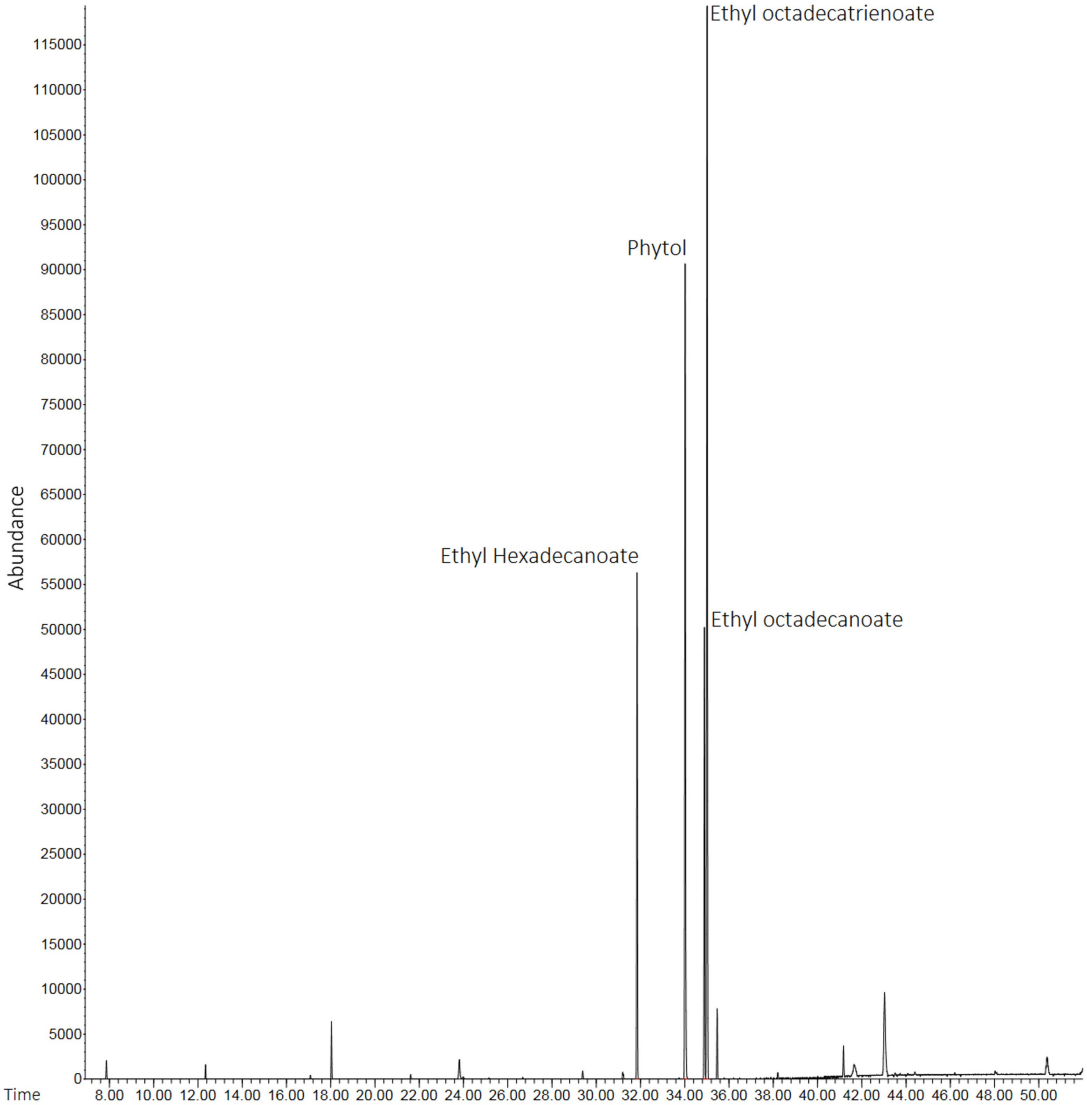
Chromatographic profiles of HEScL using gas chromatography with mass spectrometry (GC–MS) and spectral mass database (NIST 11). Time: x-axis of the gas chromatogram corresponds to retention time RT (a measure of the time taken for a solute to pass through a chromatography column) in minutes. Abundance: y-axis of the gas chromatogram (area of the peak) is a reflection of the amount of a specific analyte (relative distribution of compounds in the sample).

Lyophilized products (HEScL) were produced in the proportion of 0.163 g/ml of evaporated product (HEScL at 10%). The chromatographic profiles of HEScL showed the presence of four main phytochemical compounds, identified as ethyl hexadecanoate (relative distribution: 16.68%; retention time: 31′850″), phytol (relative distribution: 29.15%; retention time: 34′015″), ethyl octadecanoate (relative distribution: 14.78%; retention time: 34′889″) and ethyl octadecatrienoate (relative distribution: 39.69%; time retention: 35′014″), using quality indexes ranging from 91 to 98% similarity to the mass spectra reported in databases (Figure 1).

### UV–Vis spectroscopy of AgNPs-HEScL

**Figure 2:**
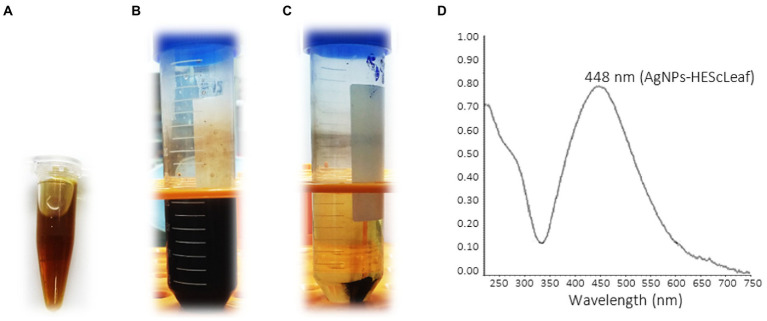
Digital photograph of the aqueous solution of the lyophilized hydroalcoholic extract of *Syzygium cumini* leaves (0.1 g/ml) **(A)**, AgNPs-HEScL solution **(B)**, centrifuged AgNPs-HEScL solution **(C)**, and ultraviolet–visible absorption spectrum of AgNPs-HEScL **(D)**.

The addition of type 1 water to the lyophilized hydroalcoholic extract of *S. cumini* leaves (0.1 g/ml) resulted in relatively opaque light green aqueous solutions of HEScLeaf (Figure 2A). The mixture of HEScLeaf solutions with 1 mM AgNO_3_ solution according to the green synthesis protocol (incubation at 90°C for 2 h) led to dark and opaque solutions (Figure 2B), indicating the development of AgNPs-HEScL (Figure 2C). The green synthesis of AgNPs-HEScL was monitored by ultraviolet–visible (UV–Vis) spectroscopy, and it detected an absorption spectrum of 448 nm (Figure 2D). In green synthesis, a total of 0.1 g of lyophilized HEScL resulted in 0.008 g of AgNPs-HEScL.

### EDX and FEG-SEM of AgNPs-HEScL

**Figure 3:**
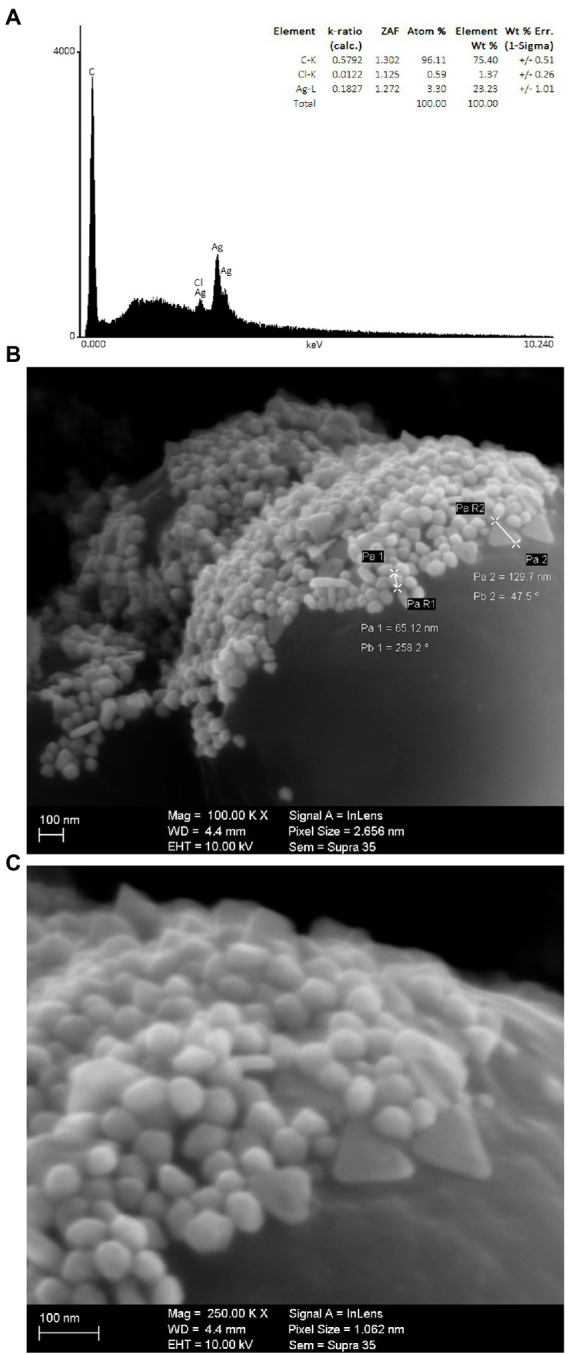
Chemical elements of AgNPs-HEScL identified by EDX **(A)**, and ultrastructural morphologies of AgNPs-HEScL obtained by FEG/SEM with magnification of 100,000× **(B)** and 250,000× **(C)** (Carl Zeiss Microscopy-Gemini Supra 35 software).

EDX analyses of AgNPs-HEScL (Figure 3A) confirmed the presence of Ag (23.23%), C (75.4%) and Cl (1.37%) ions, as well as other unidentified elements (weak signals). FEG-SEM analyses revealed a great diversity of geometrical shapes and sizes of the nanocomposites: dimensions between 65.12 and 129.7 nm, and oval, cylindrical, triangular and polyhedral shapes (Figures 3B,C).

### DLS and zeta potential of AgNPs-HEScL

**Figure 4:**
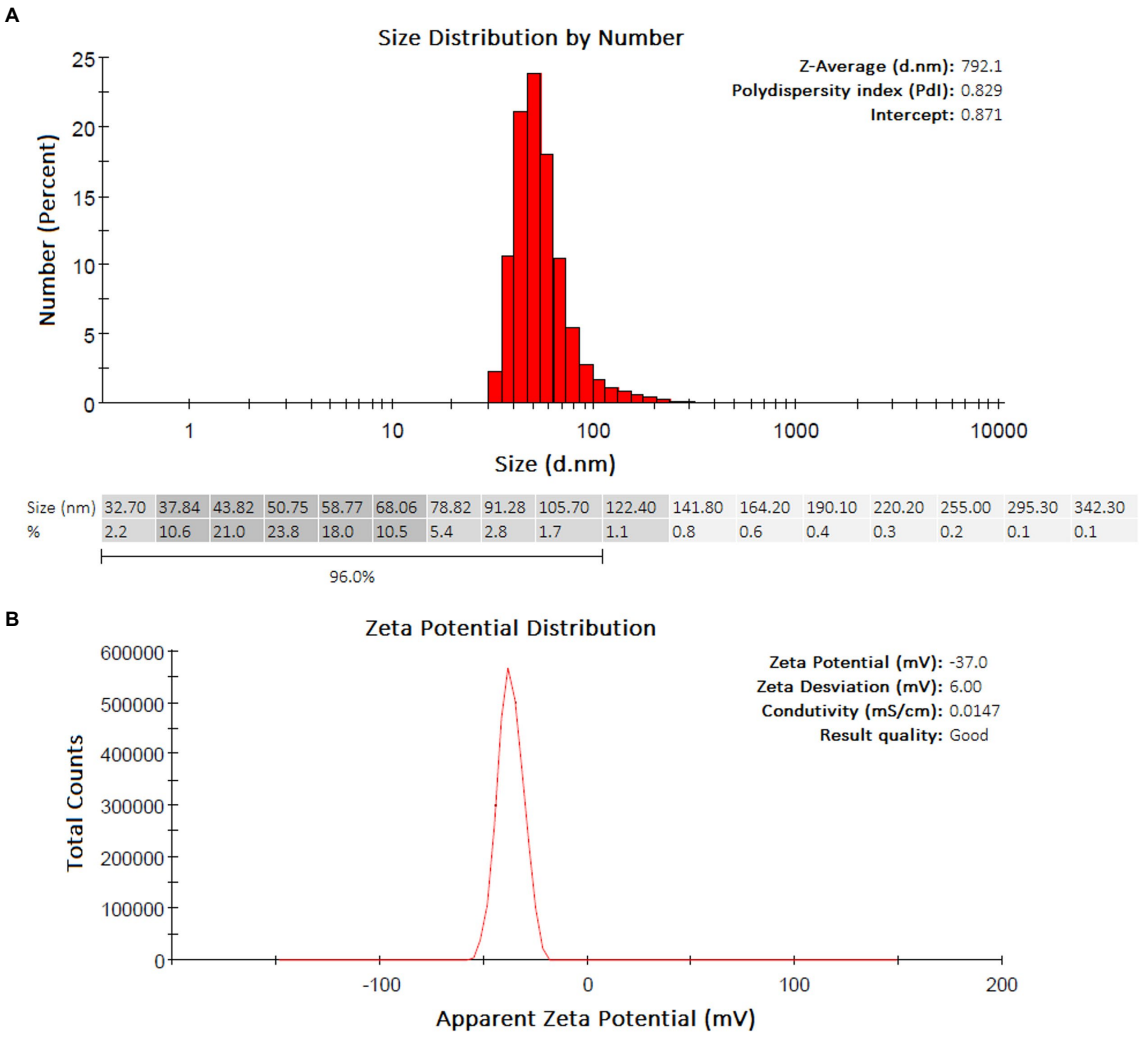
Quantitative and qualitative profiles of AgNPs-HEScL obtained by DLS **(A)** and zeta potential **(B)**.

The DLS analyses showed a predominance of groups ranging from 37.84–68.06 nm (Z-Average: 792.1 nm) and with a PdI equal to 0.829 (highly polydisperse) for AgNPs-HEScL (Figure 4A). The colloidal stability of AgNPs-plant extracts was considered highly stable, according to the analysis of zeta potential (AgNPs-HEScL: −37.0 mV, zeta deviation of 6.00 mV, conductivity of 0.0147 mS/cm; Figure 4B).

### Antimicrobial susceptibility tests of HEScL and AgNPs-HEScL

The results of antimicrobial susceptibility tests of HEScL revealed variable MIC values, depending on the microbial species. Aerobic microorganisms showed MIC values between 1,296.8 and 5,187.5 μg/ml, whereas strict and facultative anaerobic microorganisms presented MICs of 1,296.8–10,375 μg/ml and 1,296.8 μg/ml, respectively. The lowest MICs of HEScL were reported for *F. nucleatum*, *S. mutans*, *S. oralis*, *S. epidermidis* and *S. aureus* (1,296.8 μg/ml), followed by *A. naeslundii* and *C. albicans* (5,187.5 μg/ml) and *V. dispar* (10,375 μg/ml). Bactericidal activities of HEScL were observed only for *S. mutans* (1,296.8 μg/ml), *S. oralis* (2,593.7 μg/ml), *S. epidermidis* (2,593.7 μg/ml) and *S. aureus* (2,593.7 μg/ml), with bacteriostatic or fungistatic effects being observed for other species of microorganisms. Inhibition zones of different sizes were reported for all species of bacteria (halo diameters between 8 and 17 millimeters), except for the yeast *C. albicans*, with divergent values (larger or smaller species-dependent inhibition zones) in relation to CHG control (0.12% chlorhexidine digluconate; Table 1).

**Table 1:** Numerical and correlation profiles of the antimicrobial susceptibility tests of HEScL and AgNPs-HEScL involving pathogenic microorganisms.

Microorganisms	HEScL			AgNPs-HEScL			CHG	FLU	Biological response	
	MIC	MBC/MFC	M∅_h_	MIC	MBC/MFC	M∅_h_	M∅_h_	M∅_h_	*a*/(*a* + *b*)	ARA (*n*×)
Strict anaerobes										
*A. naeslundii*	5,187.5	–	12 ± 0.3	250	–	9 ± 0.0	13 ± 0.2	*na*	0.954	20.750
*V. dispar*	10,375	–	13 ± 0.0	125	–	7 ± 0.0	15 ± 0.0	*na*	0.988	83.000
*F. nucleatum*	1,296.8	–	17 ± 0.3	31.2	–	9 ± 0.3	17 ± 0.0	*na*	0.977	41.564
Facultative anaerobes										
*S. mutans*	1,296.8	1,296.8	12 ± 0.0	125	–	7 ± 0.0	13 ± 0.0	*na*	0.912	10.374
*S. oralis*	1,296.8	2,593.7	10 ± 0.0	125	–	10 ± 0.0	16 ± 0.2	*na*	0.912	10.374
Aerobics										
*C. albicans*	5,187.5	–	–	125	–	13 ± 0.1	14 ± 0.9	25 ± 0.5	0.976	41.500
*S. epidermidis*	1,296.8	2,593.7	8 ± 0.0	125	–	12 ± 0.0	17 ± 0.0	*na*	0.912	10.374
*S. aureus*	1,296.8	2,593.7	10 ± 0.7	62.5	–	12 ± 0.5	17 ± 0.0	*na*	0.954	20.749

MIC, minimum inhibitory concentration (μg/ml); MBC/MFC, minimum bactericidal concentration/minimum fungicidal concentration (μg/ml); M∅_h_, mean diameter of inhibition halos (mm); *na*, Not applicable; FLU, Fluconazole (25 μg/ml): halo > 19 mm (sensitive), halo between 14 and 19 mm (intermediate) and halo < 14 mm (resistant). CHG, Chlorhexidine digluconate (12 mg/ml). Letters *a* and *b* correspond to MIC values of HEScL and AgNPs-HEScL, respectively. ARA corresponds to antimicrobial response amplification (ARA = *a* × 1/*b*):

▪ ARA = 0.500: test substances *a* and *b* (identical concentrations) have the same biological effects (i.e., antimicrobial action: MIC).

▪ 1 > ARA > 0.500: test substances *a* (higher concentrations) and *b* (lower concentrations) have the same biological effects (i.e., antimicrobial action: MIC).

▪ 0.500 > ARA > 0.000: test substances *a* (lower concentrations) and *b* (higher concentrations) have the same biological effects (i.e., antimicrobial action: MIC).

▪ ARA equal to 0.667, 0.750, 0.800, 0.833, 0.857, 0.875, 0.889, 0.900, 0.909, …, 0.990, and 0.999 correspond to 2×, 3×, 4×, 5×, 6×, 7×, 8×, 9×, 10×, …, 100×, and 1,000× concentrations of test substance *a*, respectively, necessary to the biological effects observed in test substance *b* (1× concentration).

For AgNPs-HEScL, data from antimicrobial susceptibility tests also showed variable MIC values, depending on the microbial species. While strict and facultative anaerobic microorganisms showed MICs of 31.2–250 μg/ml and 125 μg/ml, respectively, aerobic microorganisms presented MICs between 62.5 and 125 μg/ml. Lower MICs of AgNPs-HEScL were reported for *F. nucleatum* (31.2 μg/ml), followed by *S. aureus* (62.5 μg/ml), *V. dispar*, *S. mutans*, *S. oralis*, *S. epidermidis* and *C. albicans* (125 μg/ml), and *A. naeslundii* (250 μg/ml). The effects of AgNPs-HEScL were considered bacteriostatic or fungistatic for all species of microorganisms. Inhibition zones of different sizes were also reported for all microbial species (halo diameters between 7 and 13 millimeters), with divergent values (larger or smaller species-dependent inhibition zones) in relation to CHG and FLU controls (0.12% chlorhexidine digluconate; Table 1).

The ratio of HEScL and AgNPs-HEScL, represented by MIC values, was used as a quantitative indicator of enhanced antimicrobial response among the test substances. The values of this ratio varied from 0.912 to 0.988, depending on the microbial species, that is, HEScL has microbial inhibitory effects identical to those of AgNPs-HEScL when at a concentration of 10.374× to 83× higher than the latter (or AgNPs-HEScL has microbial inhibitory effects identical to those of HEScL when at a concentration of 10.374× to 83× lower than the latter; Table 1).

### Cytotoxicity of HEScL and AgNPs-HEScL

The exposure of NOK-SI to treatments with HEScL and AgNPs-HEScL for 5 and 10 min resulted in dose-dependent cytotoxicity. Experimental cytotoxicity controls (negative controls) in NOK-SI, 0.12% chlorhexidine digluconate (CHG) and 3% hydrogen peroxide (HP), showed statistically significant differences (*p* < 0.05) between each other and cell growth (CG or positive control), that is, NOK-SI not exposed to HEScL and AgNPs-HEScL.

**Figure 5:**
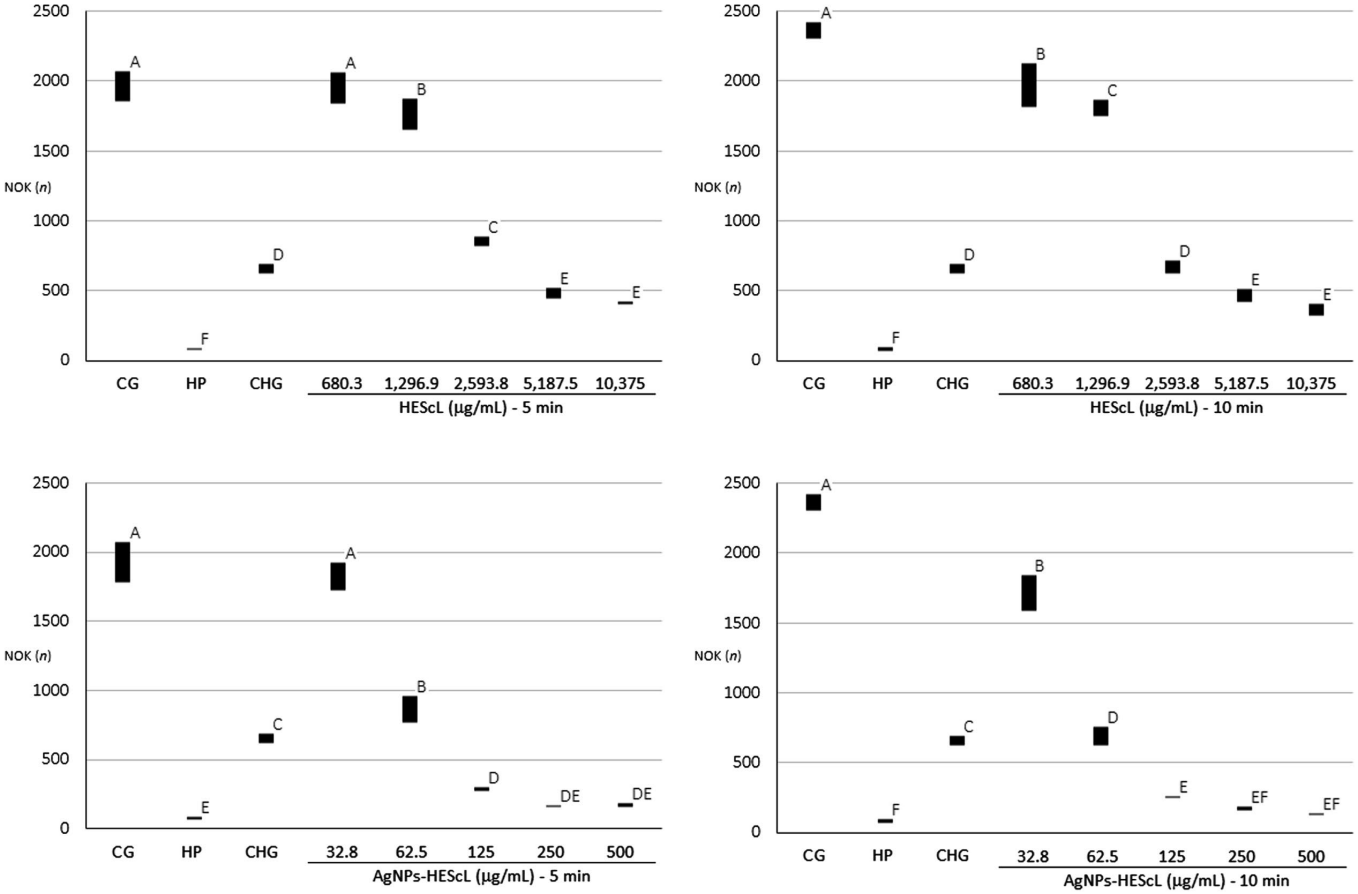
Quantitative profiles of NOK-SI obtained from those treated with different concentrations of HEScL and AgNPs-HEScL for 5 and 10 min. Means with the same letters (A, B, C, D, E, or F) are not significantly different (*p* < 0.05). CG, cell growth (positive control); HP, hydrogen peroxide (negative control); CHG, chlorhexidine digluconate (negative control).

NOK-SI treated with 680.3 μg/ml of HEScL for 5 min showed no statistically significant difference (*p* < 0.05) in relation to the CG, unlike the other concentrations of HEScL (1,296.9, 2,593.8, 5,187.5, and 10,375 μg/ml) at timepoints of 5 and 10 min (including 680.3 μg/ml for 10 min). Within 5 min, the exposure of NOK-SI to concentrations of 1,296.9 and 2,5,938 μg/ml of HEScL produced milder cytotoxic effects (*p* < 0.05) than the treatment with CHG, while at concentrations of 5,187.5 and 10,375 μg/ml of HEScL intermediate cytotoxic effects (*p* < 0.05) in relation to the experimental cytotoxicity controls of CHG and HP could be observed. Within 10 min, the treatment of NOK-SI with 680.3 and 1,296.9 μg/ml of HEScL led to milder cytotoxic effects (*p* < 0.05) than the exposure to CHG, whereas at a concentration of 2,593.8 μg/ml of HEScL the cytotoxic effect was found to be identical to that (*p* < 0.05) resulting from CHG treatment and at concentrations of 5,187.5 and 10,375 μg/ml of HEScL the cytotoxic effects (*p* < 0.05) were considered intermediate in relation to the experimental cytotoxicity controls of CHG and HP (Table 2; Figure 5).

**Table 2:** Numerical patterns of NOK-SI obtained from treatments with different concentrations of HEScL and AgNPs-HEScL for 5 and 10 min.

Testing componentsHEScL (μg/ml)	HEScL	
	5 min^A^	10 min^A^
CG	1975.7^A^ ± 121.2	2351.0^A^ ± 64.2
HP (3%)	81.2^F^ ± 5.5	80.2^F^ ± 13.0
CHG (0.12%)	660.3^D^ ± 40.5	660.3^D^ ± 40.5
680.3	1925.2^A^ ± 156.8	1955.0^B^ ± 163.3
1,296.9	1747.1^B^ ± 191.0	1825.8^C^ ± 116.5
2,593.8	839.7^C^ ± 78.0	673.3^D^ ± 71.3
5,187.5	489.0^E^ ± 52.1	451.7^E^ ± 67.3
10,375	408.7^E^±30.2	364.2^E^ ± 56.3
**Testing components** **AgNPs-HEScL (μg/ml)**	**AgNPs-HEScL**	
	**5 min**^A^	**10 min**^A^
CG	1949.4^A^ ± 161.0	2351.0^A^ ± 64.2
HP (3%)	81.2^E^ ± 5.5	80.2^F^ ± 13.0
CHG (0.12%)	660.3^C^ ± 40.5	660.3^C^ ± 40.5
32.8	1850.6^A^ ± 182.1	1682.4^B^ ± 187.1
62.5	847.4^B^ ± 140.5	677.3^D^ ± 121.9
125	283.0^D^ ± 96.2	250.7^E^ ± 12.2
250	161.6^DE^ ± 14.0	169.6^EF^ ± 21.4
500	171.1^DE^ ± 23.1	129.8^EF^ ± 13.1

Means with the same letters (A, B, C, D, E or F) are not significantly different (*p* < 0.05). Positive control: cell growth (CG). Cytotoxic controls or negative controls: HP (hydrogen peroxide) and CHG (chlorhexidine digluconate).

Despite lower concentrations of AgNPs-HEScL during the treatment of NOK-SI, their cytotoxic behavior was similar to that of HEScL. NOK-SI treated with 32.8 μg/ml of AgNPs-HEScL for 5 min showed no statistically significant difference (*p* < 0.05) in relation to the CG (positive control), unlike the other concentrations (62.5, 125, 250, and 500 μg/ml) at timepoints of 5 and 10 min (including 32.8 μg/ml for 10 min). Within 5 min, the exposure of NOK-SI to a concentration of 62.5 μg/ml of AgNPs-HEScL caused milder cytotoxic effects (*p* < 0.05) than the treatment with CHG, while at a concentration of 125 μg/ml there were intermediate cytotoxic effects (*p* < 0.05) in relation to experimental cytotoxicity controls of CHG and HP and at concentrations of 250 and 500 μg/ml the cytotoxic effects were similar to those found with HP treatment (*p* < 0.05). Within 10 min, NOK-SI treated with 32.8 μg/ml of AgNPs-HEScL showed milder cytotoxic effects (*p* < 0.05) than when treated with CHG, whereas at concentrations of 62.5 and 125 μg/ml intermediate cytotoxic effects (*p* < 0.05) in relation to the experimental cytotoxicity controls of CHG and HP could be noted and at concentrations of 250 and 500 μg/ml the cytotoxic effects were found to be identical to those resulting from HP exposure (*p* < 0.05; Table 2; Figure 5).

The ratio of HEScL and AgNPs-HEScL, represented by concentration values statistically corresponding to the (i) effects identical to positive control or cell growth (absent cytotoxicity), (ii) intermediate effects to positive and negative controls (intermediate cytotoxicity), and (iii) identical or intermediate effects to cytotoxicity controls or negative controls (cytotoxicity present), was used as a quantitative indicator of enhanced cytotoxic response among the test substances. The values of this ratio varied from 0.954 to 0.976 as follows (Table 3):

- Absent cytotoxicity: HEScL has effects identical to those of AgNPs-HEScL at a concentration of 20.7× higher than the latter (5 min), or AgNPs-HEScL has effects identical to those of HEScL at a concentration of 20.7× lower than the latter.
- Intermediate cytotoxicity: HEScL has effects identical to those of AgNPs-HEScL at a concentration of 20.7× higher than the latter (5 and 10 min), or AgNPs-HEScL has effects identical to those of HEScL at a concentration of 20.7× lower than the latter.
- Cytotoxicity present: HEScL has effects identical to those of AgNPs-HEScL at a concentration of 41.5× higher than the latter (5 and 10 min), or AgNPs-HEScL has effects identical to those of HEScL at a concentration of 41.5× lower than the latter.

**Table 3:** Correlation profiles of the cytotoxicity tests of HEScL and AgNPs-HEScL involving NOK-SI.

Comparative characteristics	LCC (5 min)		BR (5 min)		LCC (10 min)		BR (10 min)	
	HEScL (μg/ml)	AgNPs-HEScL (μg/ml)	*a*/(*a* + *b*)	CRA (*n*×)	HEScL (μg/ml)	AgNPs-HEScL (μg/ml)	*a*/(*a* + *b*)	CRA (*n*×)
▪ Effects identical to positive control or cell growth (CG): Absent cytotoxicity	680.3	32.8	0.954	20.741	–	–	–	–
▪ Intermediate effects in relation to positive and negative controls: Intermediate cytotoxicity	1,296.9	62.5	0.954	20.750	680.3	32.8	0.954	20.741
▪ Identical or intermediate effects in relation to cytotoxicity controls or negative controls (HP and CHG): Cytotoxicity present	5,187.5	125	0.976	41.500	2,593.8	62.5	0.976	41.501

LCC (Lower corresponding concentration based on Table 2; *p* < 0.05), BR (Biological response), CG (cell growth), HP (hydrogen peroxide) and CHG (chlorhexidine digluconate).

CRA corresponds to cytotoxic response amplification (CRA = *a* × 1/*b*):

▪ CRA = 0.500: test substances *a* and *b* (identical concentrations) have the same biological effects (i.e., antimicrobial action: MIC).

▪ 1 > CRA > 0.500: test substances *a* (higher concentrations) and *b* (lower concentrations) have the same biological effects (i.e., antimicrobial action: MIC).

▪ 0.500 > CRA > 0.000: test substances *a* (lower concentrations) and *b* (higher concentrations) have the same biological effects (i.e., antimicrobial action: MIC).

▪ CRA equal to 0.667, 0.750, 0.800, 0.833, 0.857, 0.875, 0.889, 0.900, 0.909, …, 0.990, and 0.999 correspond to 2×, 3×, 4×, 5×, 6×, 7×, 8×, 9×, 10×, …, 100×, and 1,000× concentrations of test substance *a*, respectively, necessary to the biological effects observed in test substance *b* (1× concentration).

### Antibiofilm susceptibility tests and SEM of AgNPs-HEScL

**Figure 6:**
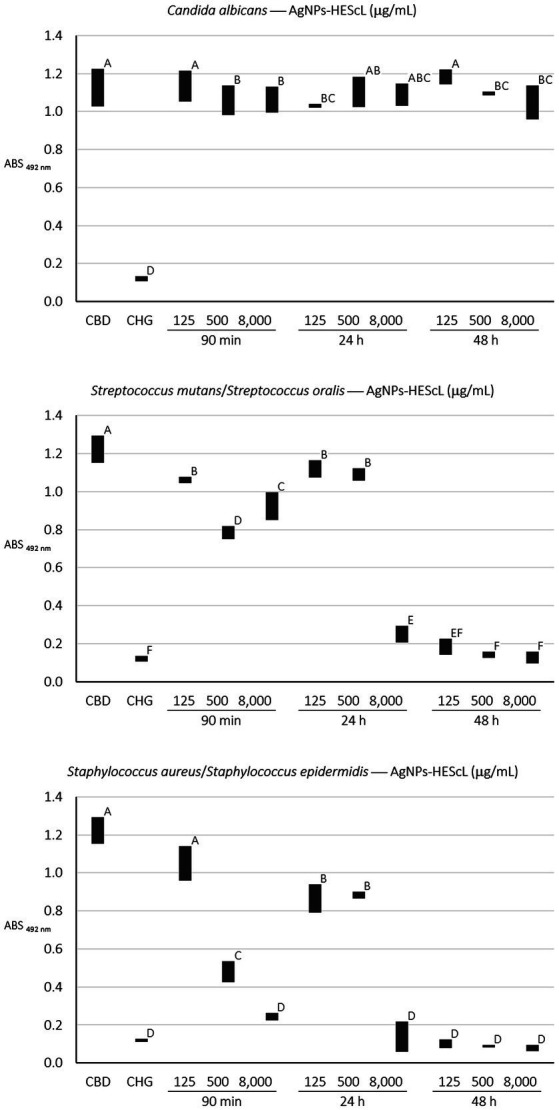
Semiquantitative profiles of the development of single (*C. albicans*) and mixed (*S. mutans* + *S. oralis* and *S. aureus* + *S. epidermidis*) biofilms obtained from treatments using different concentrations of AgNPs-HEScL for 5 min. Means with the same letters (A, B, C, D, E, or F) are not significantly different (*p* < 0.05). CBD: control of biofilm development. CHG: 0.12% chlorhexidine digluconate.

**Figure 7:**
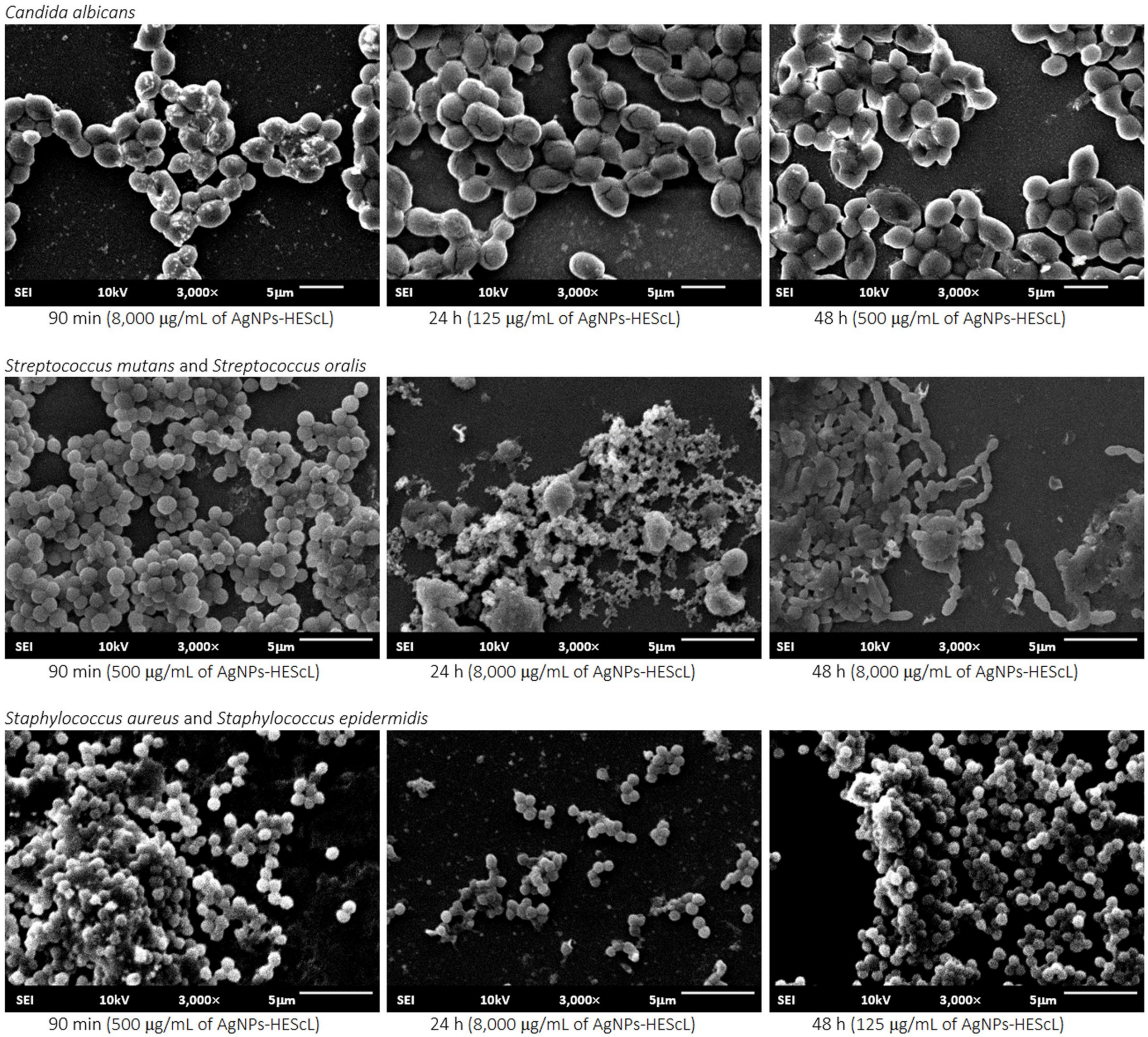
Ultrastructural and morphological images of single (*C. albicans*) and mixed (*S. mutans* + *S. oralis* and *S. aureus* + *S. epidermidis*) biofilms exposed to AgNPs-HEScL. Scanning electron microscopy (JSM-6610 LV JEOL, Tokyo, Japan) at 10 KV and 3,000× magnification.

For the formation kinetics of *C. albicans* biofilm, no statistically significant difference (*p* < 0.05) was observed between growth control and treatments with AgNPs-HEScL at concentrations of 125 μg/ml for 90 min (cell adhesion) and 48 h (advanced biofilm) and 500 μg/ml and 8,000 μg/ml for 24 h (initial biofilm). However, it was possible to note statistically significant actions (*p* < 0.05) against cell adhesion and biofilm formation of *C. albicans* when using AgNPs-HEScL at concentrations of 125 μg/ml for 24 h (initial biofilm) and 500 μg/ml and 8,000 μg/ml for 90 min (cell adhesion) and 48 h (advanced biofilm). Statistically significant differences (*p* < 0.05) were also observed between treatments with CHG (0.12% chlorhexidine digluconate) and AgNPs-HEScL at all concentrations and timepoints in comparison with growth control (GC; Table 4; Figure 6). In addition, there were ultrastructural morphological changes in the single-species biofilms (*C. albicans*) in terms of cell volume, microbial density and cell wall damage (Figure 7).

**Table 4:** Semiquantitative numerical patterns of the development of single (*C. albicans*) and mixed (*S. mutans* + *S. oralis* and *S. aureus* + *S. epidermidis*) biofilms obtained from treatments using different concentrations of AgNPs-HEScL for 5 min.

Cell adhesion/Biofilm	AgNPs-HEScL		
	8,000 μg/ml	500 μg/ml	125 μg/ml
*C. albicans*			
CBD (48 h)	1.097^A^ ± 0.186	1.097^A^ ± 0.186	1.097^A^ ± 0.186
CHG (48 h)	0.126^D^ ± 0.024	0.126^D^ ± 0.024	0.126^D^ ± 0.024
90 min	1.068^B^ ± 0.098	1.085^B^ ± 0.116	1.127^A^ ± 0.085
24 h	1.101^ABC^ ± 0.091	1.093^AB^ ± 0.089	1.031^BC^ ± 0.052
48 h	1.058^BC^ ± 0.117	1.095^BC^ ± 0.075	1.181^A^ ± 0.059
*S. oralis* + *S. mutans*			
CBD (48 h)	1.226^A^ ± 0.074	1.226^A^ ± 0.074	1.226^A^ ± 0.074
CHG (48 h)	0.118^F^ ± 0.047	0.118^F^ ± 0.047	0.118^F^ ± 0.047
90 min	0.940^C^ ± 0.071	0.780^D^ ± 0.064	1.066^B^ ± 0.032
24 h	0.243^E^ ± 0.053	1.083^B^ ± 0.037	1.131^B^ ± 0.044
48 h	0.121^F^ ± 0.034	0.146^F^ ± 0.030	0.172^EF^ ± 0.053
*S. aureus* + *S. epidermidis*			
CBD (48 h)	1.226^A^ ± 0.074	1.226^A^ ± 0.074	1.226^A^ ± 0.074
CHG (48 h)	0.118^D^ ± 0.047	0.118^D^ ± 0.047	0.118^D^ ± 0.047
90 min	0.247^D^ ± 0.061	0.496^C^ ± 0.098	1.043^A^ ± 0.102
24 h	0.115^D^ ± 0.112	0.885^B^ ± 0.073	0.847^B^ ± 0.110
48 h	0.080^D^ ± 0.073	0.089^D^ ± 0.030	0.095^D^ ± 0.037

The values correspond to means and standard deviations of absorbance obtained in the XTT reduction test. Means with the same letters (A, B, C, D, E, or F) are not significantly different (*p* < 0.05). CBD: control of biofilm development in RMPI 1640 or BHI culture medium. CHG: 0.12% chlorhexidine digluconate.

For the mixed biofilm of *S. mutans* and *S. oralis*, statistically significant differences (*p* < 0.05) between growth control and treatments with AgNPs-HEScL at all concentrations and times and treatment with CHG (0.12% chlorhexidine digluconate) were observed. Nevertheless, there was no statistically significant difference (*p* < 0.05) between (i) treatments with AgNPs-HEScL at concentrations of 500 μg/ml for 24 h (initial mixed biofilm) and 125 μg/ml at 90 min (mixed cell adhesion) and 24 h (initial mixed biofilm), or between (ii) treatments with AgNPs-HEScL at concentrations of 8,000 μg/ml, 500 μg/ml and 125 μg/ml for 48 h (advanced mixed biofilm) and treatment with CHG (0.12% chlorhexidine digluconate), or between (iii) treatments with AgNPs-HEScL at concentrations of 8,000 μg/ml for 24 h (initial mixed biofilm) and 125 μg/ml for 48 h (advanced mixed biofilm). Statistically significant actions against mixed cell adhesion and mixed biofilm formation of *S. mutans* and *S. oralis* (*p* < 0.05) were noted with AgNPs-HEScL treatments at all concentrations (125, 500, and 8,000 μg/ml) and timepoints (90 min, 24 h and 48 h). However, treatments only with AgNPs-HEScL at concentrations of 8,000 μg/ml, 500 μg/ml and 125 μg/ml for 48 h (advanced mixed biofilm) produced harmful effects (*p* < 0.05) identical to those of 0.12% chlorhexidine (i.e., action against mixed cell adhesion and mixed biofilm formation of *S. mutans* and *S. oralis*; Table 4; Figure 6). Ultrastructural morphological changes in terms of cell volume, microbial density and cell wall damage were also seen in the double-species biofilms (*S. mutans* and *S. oralis*; Figure 7).

For the mixed biofilm of *S. aureus* and *S. epidermidis*, statistically significant differences (*p* < 0.05) between growth control and treatments with AgNPs-HEScL at most concentrations and all timepoints (i.e., except for the concentration of 125 μg/ml for 90 min) and treatment with CHG (0.12% chlorhexidine digluconate) were observed. Nonetheless, there was no statistically significant difference (*p* < 0.05) between (i) treatments with AgNPs-HEScL at concentrations of 500 μg/ml and 125 μg/ml for 24 h (initial mixed biofilm), or between (ii) treatments with AgNPs-HEScL at concentrations of 8,000 μg/ml for 90 min (mixed cell adhesion) and 24 h (initial mixed biofilm) and 8,000 μg/ml, 500 μg/ml, and 125 μg/ml for 48 h (advanced biofilm). Statistically significant actions against mixed cell adhesion and mixed biofilm formation of *S. aureus* and *S. epidermidis* (*p* < 0.05) were observed with AgNPs-HEScL treatments at concentrations of 500 μg/ml and 8,000 μg/ml for 90 min and all concentrations (125, 500, and 8,000 μg/ml) for 24 h and 48 h. However, the treatments with AgNPs-HEScL at concentrations of 8,000 μg/ml for 90 min (mixed cell adhesion) and 24 h (initial mixed biofilm) and all concentrations (125, 500, and 8,000 μg/ml) for 48 h (advanced mixed biofilm) produced harmful effects (*p* < 0.05) identical to those of 0.12% chlorhexidine (i.e., action against mixed cell adhesion and mixed biofilm formation of *S. aureus* and *S. epidermidis*; Table 4; Figure 6). Ultrastructural morphological changes in terms of cell volume, microbial density and cell wall damage were also observed in the double-species biofilms (*S. aureus* and *S. epidermidis*; Figure 7).

## Discussion

The phytochemical screening of HEScL by GC–MS identified four main components: ethyl hexadecanoate (16.68%), phytol (29.15%), ethyl octadecanoate (14.78%) and ethyl octadecatrienoate (39.69%). Of these compounds, phytol was potentially associated with certain biological activities (Islam et al., 2018) and considered for potential use in a wide range of applications in the pharmaceutical and biotechnology industries. Its biological activities include: anxiolytic effects, metabolic modulators, cytotoxic agent, antioxidant, inducer of autophagy and apoptosis, antinociceptive agent and anti-inflammatory agents, and immunological and antimicrobial modulators (Islam et al., 2018). The other compounds correspond to fatty acid ethyl esters (FAEEs), i.e., products for the esterification of fatty acids and ethanol. *In vitro* and *in vivo* studies have shown the toxicity associated with FAEEs (Laposata et al., 2002). Phytochemical studies analyzing *S. cumini* leaves have also reported the presence of abundant acylated flavonol glycosides (Mahmoud et al., 2001) – quercetin, myricetin, myricitin, myricetin 3-O-4-acetyl-L-rhamnopyranoside (Timbola et al., 2002), triterpenoids (Gupta and Sharma, 1974), esterase, galloyl carboxylase (Bhatia et al., 1974) and tannin (Morton, 1987) – with medicinal properties and common use in traditional medicine (Ayyanar and Subash-Babu, 2012). In a recent phytochemical screening of this species of *S. cumini*, several other components were identified in its seeds (e.g., squalene^77.15%^, campesterol^7.35%^, tocopherol^5.36%^, octadecadienoic acid^3.45%^, hexacosane^1.62%^, hexacosanol^1.09%^ and hexadecanoic acid^0.63%^) and flowers (e.g., isooctyl phthalate^46.29%^, ethyl glucopyranoside^12.4%^, stigmasterol^4.62%^, hexadecanoic acid^4.36%^, focusterol^4.28%^, ooctamethyl octadecahydro picenone^4.25%^, octadecadiennial^4.2%^, diploptene^3.5%^, cyclolaudenol^3.32%^, ethyl linoleate^3%^, sitosterol^1.8%^, amirine^1.59%^, ethyl palmitate^1.46%^, caprolactam^1.33%^, ethyl stearate^1.14%^, tetradecenin^0.62%^, and hydroxy dihydromaltol^0.26%^), which exhibited potential biological activities including antimicrobial action (de Carvalho Bernardo et al., 2021).

Aqueous solutions of HEScL and AgNO_3_ were used for the synthesis of AgNPs-HEScL, whose dark and opaque colors indicate the presence of nanocomposites (Khan et al., 2014; de Carvalho Bernardo et al., 2021). These AgNPs-HEScL were initially monitored by ultraviolet–visible (UV–Vis) spectroscopy, and the absorption spectrum attributed to the nanoparticles was 448 nm. It was found that silver nanoparticles from plant extracts usually present absorption in a visible range from 380 to 450 nm, depending on their shapes and sizes (Sathishkumar et al., 2012; Khan et al., 2014). The results were consistent with previous studies (Sathishkumar et al., 2012; Khan et al., 2014; Kota et al., 2017; Siddiqi et al., 2018; Jalal et al., 2019; de Carvalho Bernardo et al., 2021).

EDX analyses confirmed the presence of Ag, C, and Cl ions as well as other unidentified elements (weak signals). The intense signal of C indicates that it is the element with the highest content and that it corresponds to the organic compound of HEScL adsorbed to the surface of AgNPs and to the carbon film used as a contact support. No signal of N for AgNO_3_ was observed, suggesting reduction and stability of AgNPs through HEScL (Jalal et al., 2019; Molina et al., 2019; de Carvalho Bernardo et al., 2021). Recently, the quantitative variations of elements found in AgNPs-plant extract (*S. cumini* seed and flower), especially C and Ag, has been shown to exert influence on MIC values or antimicrobial response amplification (ARA) depending on the microorganism species. Thus, it can be inferred that the antimicrobial susceptibility profiles are dependent on the microbial species and phytochemical components and elements associated with silver nanoparticles (de Carvalho Bernardo et al., 2021).

The images of AgNPs-HEScL obtained by FEG-SEM revealed different shapes (oval, cylindrical, triangular and polyhedral) and sizes. The analysis of particle size distribution by DLS showed a predominance of groups ranging from 37.84 to 68.06 and a highly polydisperse PdI (PdI: 0.829; Bhattacharjee, 2016). The particle size distribution and polydispersity index (PDI) of nanocarriers are important physical characteristics to be considered when creating pharmaceutical-grade products since they can affect the bulk properties, product performance, processability, stability and appearance of the end product (Danaei et al., 2018). Unlike AgNPs-HEScL, PdI values classified as moderately dispersive were found in silver nanoparticles synthesized from lyophilized hydroalcoholic extracts of *S. cumini* seeds (AgNPs-HEScSeed, PdI: 0.297) and flowers (AgNPs-HEScFlower, PdI: 0.358; de Carvalho Bernardo et al., 2021).

The colloidal stability of AgNPs-HEScL (−37.0 mV) was considered highly stable (Bhattacharjee, 2016). Compared to previous studies (de Carvalho Bernardo et al., 2021), this result was very similar to the AgNPs-HEScSeed (zeta potential: −36.7 mV) and higher than that reported for AgNPs-HEScFlower (zeta potential: −29.2 mV). However, the conductivity of AgNPs-HEScL (0.0147 mS/cm) was shown to be similar to the AgNPs-HEScFlower (0.0136 mS/cm) and higher than that reported for AgNPs-HEScSeed (0.00913 mS/cm). Briefly, the silver nanoparticles synthesized from different anatomical parts of *S. cumini* displayed variable physical characteristics, according to data compared (de Carvalho Bernardo et al., 2021) and obtained by the DLS and zeta potential analyses (Colloidal stability: AgNPs-HEScL ≥ AgNPs-HEScSeed > AgNPs-HEScFlower; Agglomeration: AgNPs-HEScL ≤ AgNPs-HEScSeed < AgNPs-HEScFlower; Conductivity: AgNPs-HEScL > AgNPs-HEScFlower > AgNPs-HEScSeed; Polydispersity: AgNPs-HEScL > AgNPs-HEScFlower > AgNPs-HEScSeed). It has previously been suggested that the different physical properties of these AgNPs-plant extracts can exert influence on MIC values or antimicrobial response amplification (ARA) depending on the microorganism species. Still, the same antimicrobial susceptibility profiles can be obtained from AgNPs-plant extracts exhibiting different physical properties as well as variations in phytochemical compositions, depending on the microbial species (i.e., strict anaerobic, facultative anaerobic or aerobic microorganisms; de Carvalho Bernardo et al., 2021). It is worth mentioning that the physical–chemical properties of nanomaterials (e.g., particle size, surface charge, shape, surface functionalization, impurity, crystallinity, conductivity, fluorescence, and magnetism) contribute to their behavior within biological systems (e.g., cellular uptake, toxicity and dissolution), and the adequate characterization of NPs then becomes extremely relevant due to safety concerns (Bhattacharjee, 2016).

Several factors such as nature of the plant extract, pH of the solution, reaction temperature (Rauwel et al., 2015) and extract concentration (Kota et al., 2017) can influence the size and shape distribution of nanoparticles developed by green synthesis processes. The zeta potential analysis indicated negative functional groups on the surfaces of nanoparticles (surface capping), mainly due to the behavior of the electronegative OH group. At this point, we must remember that since AgNPs are Ag^0^, they may not have electrical activity on the surface (Molina et al., 2019). Furthermore, a large number of polyphenolic compounds in plant extract might be responsible for the reduction in Ag^+^ to Ag^0^ and stabilization of AgNPs (Jalal et al., 2019).

Diverse mechanisms have been proposed to explain the antimicrobial performance of nanoparticles: oxidative stress, molecular damage (proteins and DNA) and cell death, binding to the cell membrane (electrostatic interactions), interconnection with the phosphorus-comprising compounds (DNA and sulfur-holding proteins), interaction and disruption of the microbial cell wall, and changes in the respiratory cycle, DNA replication blockage and cell division (Mirzajani et al., 2011; Nasrollahi et al., 2011; Prabhu and Poulose, 2012; Bondarenko et al., 2013; Hassan et al., 2013; Reidy et al., 2013; Ojo et al., 2018; Mittal et al., 2019; Asghar et al., 2020).

HEScL showed antibacterial and antifungal action with inhibitory concentrations dependent on the microbial species evaluated. The species of *F. nucleatum*, *S. mutans*, *S. oralis*, *S. epidermidis* and *S. aureus* were sensitive to lower concentrations of HEScL (1,296.8 μg/ml), whereas *A. naeslundii*, *C. albicans* and *V. dispar* were inhibited by higher concentrations (5,187.5–10,375 μg/ml). HEScL showed bactericidal action for *S. mutans*, *S. oralis*, *S. epidermidis* and *S. aureus*, with an identical concentration of MIC, or one above. HEScL produced medium-sized microbial inhibition zones ranging from 8 to 17 mm in diameter, which were smaller than the those created by the respective controls of CHG (0.12% chlorhexidine digluconate), except for *F. nucleatum* (identical diameter) and *C. albicans* (absence of inhibition zone). AgNPs-HEScL also showed an antimicrobial action against all tested species. Although considered bacteriostatic or fungistatic, AgNPs-HEScL presented significantly smaller MICs when compared to HEScL (31.2–250 μg/ml). The inhibition zones produced by AgNPs-HEScL had diameters from 7 to 13 mm, which were smaller than those created by the respective controls of CHG or FLU. These results suggest a potential broad-spectrum antimicrobial effect of HEScL and AgNPs-HESc, especially when considering strict anaerobic, facultative and aerobic microbial agents whose MIC values vary with the microbial species. For the first time in the literature, this study reports the antimicrobial action of AgNPs-HEScL, whose inhibitory and bacteriostatic/fungistatic effects were produced at a concentration of >10× to 83× lower than HEScL.

Similar results were observed in previous studies with lyophilized hydroalcoholic extract of *S. cumini* seed and flower (HEScSeed and HEScFlower) and their silver nanoparticles (AgNPs-HEScSeed and AgNPs-HEScFlower) for the same microbial species (de Carvalho Bernardo et al., 2021): (i) bacteriostatic/fungistatic or bactericidal effects for HEScSeed and HEScFlower and MIC of 648.4–2,593.7 μg/ml and 1,296.8-5,187.5 μg/ml, respectively, depending on the microbial species (medium-sized microbial inhibition zones ranging from 7 to 18 mm in diameter); and (ii) bacteriostatic/fungistatic effects for AgNPs-HEScSeed and AgNPs-HEScFlower and MIC of 31.2–2,000 μg/ml and 31.2–250 μg/ml, respectively, depending on the microbial species (medium-sized microbial inhibition zones ranging from 7 to 12 mm in diameter, except for *C. albicans* tested with AgNPs-HEScSeed). The antimicrobial action of AgNPs-HEScSeed and AgNPs-HEScFlower was observed at a concentration of 5× to 166× lower than plant extracts, except for *C. albicans* tested with AgNPs-HEScSeed. This points to a potential broad-spectrum antimicrobial effect of HEScSeed, HEScFlower, AgNPs-HEScSeed and AgNPs-HEScFlower, especially when considering strict anaerobic, facultative and aerobic microbial agents whose MIC values vary with the microbial species. The antimicrobial susceptibility tests using disk diffusion revealed medium-sized microbial inhibition zones ranging from 7 to 18 mm in diameter for plant extracts (HEScSeed and HEScFlower) and from 7 to 12 mm in diameter for their nanoparticles (AgNPs-HEScSeed and AgNPs-HEScFlower), depending on the microbial species, except for *C. albicans* tested with AgNPs-HEScSeed (de Carvalho Bernardo et al., 2021). Antimicrobial assays using plant extracts have shown absence of correlation between agar diffusion and microdilution methods, with several factors being potentially responsible for the lack of reproducibility among the methods, such as physicochemical characteristics of plant extracts, culture media, pH, oxygen availability, inoculum and incubation conditions (Boriollo et al., 2020).

Bacterial and yeast species can adhere and form biofilms on a variety of scaly oral surfaces, e.g., epithelial cells, as well as on a variety of non-scaly surfaces, e.g., teeth, implants, or removable dentures. The formation of biofilms on biomaterials, such as oral prostheses, has important clinical repercussions due to their greater resistance to antimicrobial therapy and host immune responses. Moreover, these biofilms are a common source of reinfection, causing health risks and economic losses. Therefore, research involving alternative therapy capable of eliminating or reducing the colonization of medical and/or dental biomaterials, and consequently the different forms of stomatological infections, could greatly contribute to the development of substitute toothpastes for prophylaxis and hygiene (Bassi and Boriollo, 2020). Additionally, biofilm study models may contain cultivable species representative of ecosystems that colonize the oral mucosa, teeth, dentures, and peri-implantitis pockets. Such models can combine reference microbial strains, wild-type strains or mutant strains, and the number of species can range from 2 to 11 strains (Chevalier et al., 2018). For such reason, adequate *in vitro* assays can help improve oral care products, drug formulations or the composition of foods, beverages and oral nutritional supplements (Chevalier et al., 2018), in addition to enabling the development of new therapeutic techniques for the treatment of oral infections and denture stomatitis (Bassi and Boriollo, 2020). The current research evaluated the effects of AgNPs-HEScL (125, 500, and 8,000 μg/ml) on cell adhesion (90 min) and single and mixed biofilms (24 and 48 h) at one exposure time of the test substance (5 min). Partial effects of AgNPs-HEScL (i.e., effects significantly different from positive control or control for inhibition of biofilm formation kinetics: 0.12% chlorhexidine digluconate) on the formation kinetics of biofilm were observed in the following situations: cell adhesion (500–8,000 μg/ml), initial biofilms (125 μg/ml) and advanced biofilms (500–8,000 μg/ml) of *C. albicans*; cell adhesion (125–8,000 μg/ml) and initial biofilms (125–8,000 μg/ml) of *S. mutans* + *S. oralis*; and cell adhesion (500 μg/ml) and initial biofilms (125–500 μg/ml) of *S. aureus* + *S. epidermidis*. Effects of nanoparticles equivalent to the positive control (chlorhexidine digluconate) were detected in advanced biofilms (125–8,000 μg/ml) of *S. mutans* + *S. oralis* (concentration of 1× to 64× higher than MIC), and cell adhesion (8,000 μg/ml), initial biofilms (8,000 μg/ml) and advanced biofilms (125–8,000 μg/ml) of *S. aureus* + *S. epidermidis* (concentration of 1× to 64×*^S. epidermidis^* or 2× to 128×*^S. aureus^* higher than MIC). In addition, the existence of *S. mutans* and *S. oralis* or *S. aureus* and *S. epidermidis* in double-species biofilm was confirmed by the screening assay using controls of biofilm development and selective and differential culture media. The bacterial biofilms had their different surfaces scraped off, and were suspended in buffer, serially diluted, spread in plate and properly incubated: TYCSB agar for *S. mutans* (TYCSB Agar Base; Tryptone Yeast Extract Cystine w/Sucrose & w/o Bacitracin Agar Base, Cat.# M1975, HiMedia Laboratories Pvt. Ltd. Mumbai, India), blood agar for *S. oralis* (Wang et al., 2020), CHROMagar™ Staphylococcus (CHROMagar™ and Rambach™, Paris, France) and coagulase test for *S. aureus* and *S. epidermidis* (Boriollo et al., 2018). All species were qualitatively detected experimentally (data not shown).

Infections associated with microbial biofilms and the growing phenomenon of antimicrobial resistance have been considered a serious public health problem (Del Pozo, 2018) and at the same time driven the discoveries and development of therapeutic bioactives against microbial pathogenesis. Some studies have demonstrated the antifungal and antibiofilm actions of aqueous or hydroalcoholic extract and essential oil of *S. cumini* leaves, especially for the yeast *C. albicans* (Oliveira et al., 2007; Höfling et al., 2010; dos Santos et al., 2012; Pereira et al., 2016) and some gram-positive and gram-negative bacteria (Satish et al., 2008; Ugbabe et al., 2010; Baliga et al., 2011; Ojo et al., 2018; Gupta et al., 2019; Mittal et al., 2019). Metal nanoparticles synthesized by *S. cumini* leaves have shown potential applications in the medical, pharmaceutical, agronomic and food areas (Asghar et al., 2020). The antimicrobial effects of Fe, Cu and Ag-NPs synthesized from *S. cumini* leaves was observed in methicillin-(MRSA) and vancomycin-resistance (VRSA) *Staphylococcus aureus* infectious strains and toxigenic strains of *Aspergillus flavus* and *A. parasiticus*. The Ag-NPs produced the better antimicrobial effects for MRSA and VRSA (inhibition zone: 18–20 mm; MIC: 8 μg/ml), antifungal effects (10–100 μg/ml) and reduced production of aflatoxins contamination (Asghar et al., 2020). These findings were promising for *S. aureus* and different than those reported for AgNPs-HEScSeed (125 μg/ml) and AgNPs-HEScFlower (250 μg/ml; de Carvalho Bernardo et al., 2021) or AgNPs-HEScL (62.5 μg/ml). The divergence of these results could be partially explained by the intrinsic characteristics of the microbial strains (i.e., MRSA, MSSA, VRSA and VSSA), the geographic location of the plant species and the methods used for the processing of extracts and synthesis of nanoparticles.

The *in vitro* cytotoxicity of HEScL and AgNPs-HEScL was evaluated in normal oral keratinocytes spontaneously immortalized (NOK-SI) at different concentrations of the test substances and two timepoints. The results potentially suggest dose- and time-dependent cytotoxic effects of HEScL and AgNPs-HEScL. However, absent cytotoxicity was observed in NOK-SI treated with HEScL at an initial concentration of 680.3 μg/ml for 5 min, while absent cytotoxicity was noted in NOK-SI exposed to AgNPs-HEScL at an initial concentration of 32.8 μg/ml for 5 min. This study presents toxicological information about the lyophilized ethanolic extract of *S. cumini* leaves, including their metallic nanoparticles, and adds scientific values to incipient studies found in the literature. Samples of the lyophilized hydroalcoholic extract of *S. cumini* leaves were experimentally analyzed for their cytotoxic activity in human cells (human keratinocyte HaCat) and rodent cells (murine macrophage RAW264.7) to provide evidence for clinical application (Pereira et al., 2016). The extract of *S. cumini* did not show cytotoxicity on macrophage cells at all tested concentrations (10–200 μg/ml). However, regarding human keratinocytes, the threshold cytotoxicity (i.e., limit concentration that does not affect cell viability) of this extract was 100 μg/ml, a value that was considered lower than the antimicrobial concentrations (i.e., planktonic cells and biofilms of *C. albicans*; Pereira et al., 2016). *In vitro* cytotoxicity tests with ethyl acetate (EA) fraction of the methanolic extract of *S. cumini* L. leaves in rodent cells (murine monocytic macrophage cells RAW 264.7) did not reveal any significant effect on cell viability and morphology at all tested concentrations (from 100 to 1,000 μg/ml; Gupta et al., 2019). According to another *in vitro* study (Ribeiro et al., 2014), the hexane extract of *S. cumini* has low toxicity in rodent macrophages, with a IC_50_ value of 31.64 μg/ml. In another *in vivo* study (Silva et al., 2012), the hydroalcoholic extract of *S. cumini* leaves did not present acute or chronic toxic effects on rodents treated orally (LD_50_ value of 489 mg/kg).

The radioprotective activity of the extract (petroleum ether-chloroform and dichloromethane-methanol) produced with *S. cumini* leaves (50 mg/kg of body weight) was evaluated in terms of prevention of radiation-induced micronuclei development in cultured mouse splenocytes exposed to different doses of gamma radiation (Jagetia and Baliga, 2002). The oral administration of this extract for five consecutive days before exposure to different doses of gamma radiation reduced the frequency of micronuclei and protected splenocytes against complex DNA damage. The radioprotective activity of *S. cumini* can be attributed to an interaction of several mechanisms, including elimination of free radicals, interruption of lipid peroxidation induced by radiation, regulation of antioxidant mechanisms, inhibition of mRNA activation of NF-κB and COX-II, regulation of DNA polymerase and efficient repair of cell genome damage (Jagetia and Baliga, 2002). The effect of lyophilized aqueous extract of *S. cumini* leaves on carbon tetrachloride-induced hepatotoxicity in male albino Wistar rats was investigated in a study (Moresco et al., 2007). The group of animals previously treated with the extract (900 mg/kg/7 days) and a single intraperitoneal dose of carbon tetrachloride presented significantly lower activities of serum aspartate aminotransferase (AST) and alanine aminotransferase (ALT) when compared to the group of animals that received carbon tetrachloride only. Even though this was the first study to demonstrate the hepatoprotective effect of *S. cumini* leaves in association with a test drug, it did not show any evidence of the effect of this plant when administered alone (Moresco et al., 2007).

The use of ethanolic extract of *S. cumini* leaves as a supplement in mice ration (50 μg/g body weight/day) was evaluated for its potential to prevent adverse effects caused by arsenic (10 μg/g body weight/day) in experimental mice (Barai et al., 2017). Animals exposed to arsenic with extract supplementation were partially protected from weight loss when compared to the group exposed to arsenic only, evidencing the effectiveness of *S. cumini* leaves against arsenic-induced toxicity, in addition to their suggestive non-toxic nature. This supplementation also blocked hepatomegaly, splenomegaly and kidney enlargement, significantly restoring organ weights to their almost normal volumes, and consequently indicating the potential therapeutic effect of *S. cumini* leaves against arsenic toxicity. However, its ability to reduce arsenic deposition in body organs was not considered significant. Also, the elevation of toxicological blood parameters [i.e., activities of alkaline phosphatase (ALP), alanine aminotransferase (ALT) and lactate dehydrogenase (LDH)] was considerably reduced when the extract was co-administered with arsenic although *S. cumini* did not significantly reduce the high serum levels of glucose and uric acid induced by arsenic (Barai et al., 2017).

Toxicological studies of other anatomical parts of *S. cumini* were also investigated. The antigenotoxic potential of the methanolic extract of *S. cumini* seeds was determined by chromosomal aberration and micronucleus tests in mouse bone marrow, sperm abnormality test and oxidative stress (Tripathi et al., 2013). The oral administration of doses ranging from 100 to 1,500 mg/kg of the extract did not induce drug-related toxicity in animals, as evidenced by survival of 100% of treated animals, in addition to lack of behavioral changes, breathing pattern and neuromuscular coordination. Pretreatment with *S. cumini* extract (200 mg/kg) resulted in aberrant metaphases compatible with data obtained from the negative control animals, significantly reducing the frequency of structural chromosomal aberrations induced by cyclophosphamide. The animals treated with cyclophosphamide showed reduced mitotic index (ratio between cell cycle progression to inhibition of cell proliferation), while the animals pretreated with *S. cumini* extract showed significantly improved mitotic activity. The administration of *S. cumini* extract (200 mg/kg) alone also indicated absence of genotoxicity and cytotoxicity in the bone marrow of mice and ensured protection against genotoxicity and cytotoxicity in bone marrow induced by cyclophosphamide, according to data from the frequency of micronucleated polychromatic erythrocytes (MNPCEs) and the ratio between polychromatic erythrocytes and normochromic erythrocytes (PCE/NCE ratio; Tripathi et al., 2013). Pretreatment with *S. cumini* extract at different dose levels also prevented morphological changes induced by cyclophosphamide and sperm abnormality rates compatible with the negative control. Therefore, considering the above the methanolic extract of *S. cumini* seeds inhibited the *in vivo* genotoxicity of cyclophosphamide, supporting the idea that its protective effect against genotoxicity and oxidative stress [i.e., it increases the content of reduced glutathione (GSH), enhances superoxide dismutase (SOD) and catalase (CAT) activities and reduces the level of malondialdehyde (MDA) against the action of cyclophosphamide in the liver of mice] results from the combined and synergistic effects of phytochemical compounds, whose combined actions can bring health benefits (Tripathi et al., 2013).

Furthermore, the protective effect of the methanolic extract of *S. cumini* seeds on the arsenic-induced genotoxicity and hepatotoxicity was also evaluated in albino Wistar rats (Kumar and Thakur, 2018). The oral administration of this extract led to normalization of biochemical variables altered by the action of arsenic [e.g., serum aspartate aminotransferase (AST), alkaline phosphatase (ALP), alanine aminotransferase (ALT) and total concentrations of protein and bilirubin], and significantly restored the activity of enzymatic and non-enzymatic antioxidants of cells [e.g., lipid peroxidation (LPO), superoxide dismutase (SOD), catalase (CAT), reduced glutathione (GSH)]. According to the results from the comet assay, the administration of this extract did not result in significant genotoxicity, and the co-administration of the extract and arsenic led to a considerable decrease in DNA damage of lymphocytes in a dose-dependent manner (Kumar and Thakur, 2018). It can then be inferred that the extract of *S. cumini* seeds offers powerful antioxidant activity and elimination of free radicals due to the presence of active compounds, especially flavonoids (e.g., myricetin, quercetin and kaempferol) and polyphenol ellagic acid (Singh et al., 1988; Tanaka et al., 1988), which may be responsible for its antitoxic and antigenotoxic effects induced by arsenic.

## Conclusion

HEScL and AgNPs-HEScL were found to have antimicrobial action against pathogens of medical and dental interest (strict anaerobes: *A. naeslundii*, *F. nucleatum* and *V. dispar*; facultative anaerobes: *S. mutans* and *S. oralis*; aerobes: *C. albicans*, *S. epidermidis*, and *S. aureus*), with species-dependent minimal inhibitory concentrations. Species-dependent bactericidal effects were observed in the treatment with HEScL. AgNPs-HEScL showed exclusively bacteriostatic and fungistatic effects, but at significantly lower concentrations than HEScL. In addition, AgNPs-HEScL exhibited potential partial action against the development kinetics of single (*C. albicans*) and mixed (*S. mutans*/*S. oralis* and *S. aureus*/*S. epidermidis*) biofilms, depending on the dose and timepoint. *In vitro* cytotoxicity of HEScL and AgNPs-HEScL occurred in a dose- and time-dependent manner in NOK-SI at lower concentrations or very close to minimum inhibitory concentrations of planktonic cells, depending on the microbial species.

HEScL had a low proportion of phytol when compared to the content of fatty acid ethyl esters, which in turn were shown to have an antimicrobial action and adversely influence the development kinetics of single and mixed biofilms (i.e., partial effect against cell adhesion and initial and mature antibiofilm). On the other hand, fatty acid ethyl esters may have been responsible for *in vitro* cytotoxicity in normal oral keratinocytes spontaneously immortalized. AgNPs-HEScL produced from plant extract had different morphologies, sizes, organic compounds, stability and electronegativity (capping) – characteristics that contribute to their biological effects.

## Data availability statement

The raw data supporting the conclusions of this article will be made available by the authors, without undue reservation.

## Author contributions

WB, MB, and DS conceptualized the study. WB, MB, and FM designed the methodology and curated the data. WB, MB, MO, and FM performed formal analysis. WB, MB, CT, MO, and JS conducted the investigation. WB and MB prepared the original draft. WB, MB, FM, and DS reviewed and edited the manuscript. MB and DS acquired funding. All authors contributed to the article and approved the submitted version.

## Funding

This research was supported by Rede Mineira de Ensaios Toxicológicos e Farmacológicos de Produtos Terapêuticos (REDE MINEIRA TOXIFAR), Fundação de Amparo à Pesquisa do Estado de Minas Gerais (FAPEMIG process no. RED-00008-14) and Fundação de Amparo à Pesquisa do Estado de São Paulo (FAPESP process no. 2022/05454-9).

## Conflict of interest

The authors declare that the research was conducted in the absence of any commercial or financial relationships that could be construed as a potential conflict of interest.

## Publisher’s note

All claims expressed in this article are solely those of the authors and do not necessarily represent those of their affiliated organizations, or those of the publisher, the editors and the reviewers. Any product that may be evaluated in this article, or claim that may be made by its manufacturer, is not guaranteed or endorsed by the publisher.
